# A Computational Approach to Identify Novel Protein Targets Uncovers New Potential Mechanisms of Action of Mirtazapine *S*(+) and *R*(−) Enantiomers in Rett Syndrome

**DOI:** 10.1111/jnc.70093

**Published:** 2025-05-26

**Authors:** Ottavia Maria Roggero, Nicolò Gualandi, Viviana Ciraci, Vittoria Berutto, Emanuele Carosati, Enrico Tongiorgi

**Affiliations:** ^1^ Cellular and Developmental Neurobiology Lab—Department of Life Sciences University of Trieste Trieste Italy; ^2^ Computational Genomics Laboratory Scuola Internazionale Superiore di Studi Avanzati (SISSA) Trieste Italy; ^3^ Department of Medicine University of Udine Udine Italy; ^4^ Department of Chemical and Pharmaceutical Sciences University of Trieste Trieste Italy

**Keywords:** antidepressants, BioGPS, drug‐protein docking, intellectual disability, mirtazapine, Rett syndrome, target deconvolution

## Abstract

Rett syndrome (RTT) is a progressive neurodevelopmental disorder that affects approximately 1:10000 newborn girls and is primarily caused by mutations in the X‐linked gene *MECP2*. Due to reduced brain monoamine levels in RTT, antidepressants have been explored as potential therapies. In previous studies, we demonstrated that the antidepressant mirtazapine (MTZ) alleviates symptoms in *Mecp2*‐mutant mice and RTT adult patients. However, the mechanism of action of MTZ, a racemic mixture that binds to multiple receptors, remains unclear. This study introduces a computational approach to screen the “human pocketome,” comprising over 25 K ligand‐bound pockets derived from more than 210 K human protein structures available in the RCSB Protein Data Bank, aiming to identify binding pockets with high affinity for each MTZ enantiomer. Novelty concerns the approach to compare the two enantiomers of MTZ to other drugs experimentally determined as inactive for RTT. This approach introduces a new metric, the ZZscore, which ranks tested proteins and pockets based on their degree of interaction with the tested drugs. This enables the identification of potential drug‐protein interactions relevant to the disease and/or phenotypic traits under study. Initial relaxed settings and thresholds parameters suggested over 30 potential targets, among which the RASH/SOS1 complex, but in vitro experiments on cultured hippocampal neurons from *Mecp2*‐KO mice excluded any MTZ effect on it. Thus, we refined the procedure with more stringent parameters and identified 16 protein targets for *S*(+)MTZ and 14 for *R*(−)MTZ, with 5 common targets. Pathway enrichment analysis revealed 25 pathways for *S*(+)MTZ and 24 for *R*(−)MTZ, with 11 common pathways, many related to MeCP2 functions disrupted in RTT, such as epigenetic chromatin regulation, intracellular signaling, energy metabolism, cholesterol and lipid metabolism, and catecholamine biosynthesis. Overall, the presented computational modeling strategy for target identification allowed us to hypothesize new mechanisms of action for the two MTZ enantiomers.

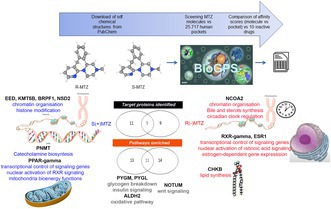

## Graphical abstract

Computational pipeline to identify potential drug targets and pathways for Rett syndrome, by analyzing ligand‐bound pockets and predicted drug affinities.

**Abbreviations**
3D‐QSAR: three‐dimensional quantitative structure–activity relationship
5‐HT: 5‐Hydroxytryptamine (Serotonin)
5‐HT3: Serotonin 3 Receptor
Å: Angström
Abs: antibodies
AKT1: Protein kinase B (Akt)
ALDH2: Aldehyde Dehydrogenase 2
Ara‐C: D‐arabinofuranoside
BCL2: B‐cell lymphoma 2
BDNF: brain‐derived neurotrophic factor
BioGPS: Software for drug design
BRD2: bromodomain containing 2
BRD4: bromodomain containing 4
BSA: bovine serum albumin
cDNA: complementary DNA
CHKB: choline kinase beta
CRY: Probe from GRID and BioGPS software, for hydrophobic regions
DA: Dopamine
DIV: days in vitro
DMEM: Dulbecco's modified eagle medium
DMSO: dimethyl sulfoxide
EP: endpoints/neuron
ESR1: estrogen receptor 1
EZH2: enhancer of zeste homolog 2
FBS: fetal bovine serum
FKBP5: FK506 binding protein 5
FLAP: software for drug design (Fingerprints for Ligands and Proteins)
GlobSum: Global Sum score from BioGPS software
GPCRs: G‐protein coupled receptors
GRID: software for drug design
GRIP1: glucocorticoid receptor‐interacting protein 1
GSEA: gene set enrichment analysis
GSK3: glycogen synthase kinase 3
GSK3B: Glycogen synthase kinase 3 beta
H1: histamine receptor 1
HBSS: Hank's balanced salt solution
HDAC6: histone deacetylase 6
HIF1A: hypoxia‐inducible factor 1‐alpha
HMOX1: heme oxygenase 1
HRAS: Harvey rat sarcoma viral oncogene homolog
KAT2B: K(lysine) acetyltransferase 2B
KDM5A: lysine‐specific demethylase 5A
KMT5B: lysine methyltransferase 5B
KO: knock‐out
LPA1: Lysophosphatidic acid receptor 1
MAP 2: microtubule‐associated protein 2
MECP2: Methyl‐CpG Binding Protein 2
MIF: molecular interactions fields (from GRID software)
MoA: mechanism of action
MTZ: mirtazapine
NA: noradrenaline
NCOA2: nuclear receptor coactivator 2
NCOR1: nuclear receptor corepressor 1
NCOR2: nuclear receptor corepressor 2
NES: Normalized Enrichment Score
NES: Normalized Enrichment Score
NPC1: Niemann‐Pick C1
NQO1: NAD(P)H dehydrogenase quinone 1
NTRK1: neurotrophic receptor tyrosine kinase 1
PCR: polymerase chain reaction
PDB: RCSB Protein Data Bank
PDE4D: phosphodiesterase 4D
p‐ERK1/2: phosphorylated extracellular signal‐regulated kinase 1/2
PFA: paraformaldehyde
PPARG: peroxisome proliferator‐activated receptor gamma
PYGL: liver form of glycogen phosphorylase
PYGM: muscle form of glycogen phosphorylase
R(−)MTZ: R(−) enantiomer of mirtazapine
RAS/MAPK: Ras/Mitogen‐Activated Protein Kinase
RASH: Harvey rat sarcoma viral oncogene homolog
RCSB: Research Collaboratory for Structural Bioinformatics
RRID: Research Resource Identifier
RTT: Rett syndrome
RXR: Retinoid X Receptor
RXRG: Retinoid X Receptor Gamma
S(+)MTZ: S(+) enantiomer of mirtazapine
SDF: structure data file
SDS‐PAGE: Sodium Dodecyl Sulfate Polyacrylamide Gel Electrophoresis
SEM: standard error of the mean
SOS1: Son Of Sevenless homolog 1
SRC‐2: Steroid Receptor Coactivator 2
TDL: total dendritic length
t‐ERK: Total‐ERK
TIF2: Transcriptional Intermediary Factor 2
TrkB: Tropomyosin receptor kinase B
Uniprot: universal protein resource
WT: wild‐type
Zscore: standardized score in statistical analysis
Zscore_mol: Zscore molecule
Zscore_poc: Zscore pocket
ZZscore: sum of Zscore_poc and Zscore_mol
α2: Alpha‐2 Adrenergic Receptor

## 1 Introduction

Rett syndrome (RTT) is a postnatal, progressive neurodevelopmental disorder affecting approximately 1 in 10 000 girls worldwide (Chahrour and Zoghbi 2007; Williamson and Christodoulou 2006). In 95% of cases, RTT is due to loss‐of‐function mutations in the methyl CpG‐binding protein 2 (*MECP2*) gene. The primary symptoms of the disease include developmental arrest, loss of language and motor skills, seizures, breathing irregularities, brain atrophy, and behavioral problems with features associated with autism (Amir et al. 1999; Dragich et al. 2000; Mount et al. 2002; Glaze et al. 2010; Cheng et al. 2019; Fonzo et al. 2020; Singh et al. 2021). The main morphological hallmark of the pathology is neuronal atrophy, highlighted by histological analysis of postmortem brains of RTT patients, which revealed smaller and more densely packed neurons with reduced dendritic complexity (Armstrong et al. 1995, 1998; Belichenko et al. 1996). To date, gene therapies are in clinical trials, and one pharmacological treatment has been recently approved (in March 2023, trofinetide was approved for medical use in RTT in the United States), but there is still no definitive cure for RTT (Cordeiro 2023; Powers et al. 2023).

In the search for new therapeutic approaches for RTT, antidepressants have been considered, based on the evidence of reduced brain levels of the monoamines serotonin (5‐HT), noradrenaline (NA), and dopamine (DA) in RTT patients and *Mecp2‐*KO mouse models in which the gene responsible for RTT was deleted (Samaco et al. 2009; Temudo et al. 2009). Desipramine was the first example of an antidepressant to be studied in RTT. It is a noradrenaline reuptake inhibitor that was able to improve breathing and lifespan in *Mecp2*‐null mice (Roux et al. 2007). Unfortunately, the phase II clinical trial was stopped because of cardiovascular side effects (Mancini et al. 2018). Although desipramine was not confirmed in patients, the overall results support the possible use of antidepressants in RTT.

Mirtazapine (MTZ) was the second antidepressant tested in RTT. It is a dual‐acting, both noradrenergic and serotoninergic (NaSSA) antidepressant that acts mainly presynaptically as an antagonist of α2‐autoreceptors and α2‐heteroreceptors and has a safer profile compared to desipramine because it has no anticholinergic and cardiac side effects (Burrows and Kremer 1997; Hartmann 1999; Szegedi and Schwertfeger 2005; Montgomery et al. 1998). Currently, MTZ is used to treat major depressive disorder, but it has also been used off‐label to treat autistic children because of its sedative, anxiolytic, and appetite‐stimulating effects (Doyle and McDougle 2012a, 2012b; Persico et al. 2021; McDougle et al. 2022). It is also used in the treatment of several comorbidities, including insomnia, panic disorder, posttraumatic stress disorder, obsessive‐compulsive disorder, generalized anxiety disorder, social anxiety disorder, and headaches and migraines (Jilani et al. 2023). In all these cases, MTZ is used as a mixture of the two enantiomers, namely *S*(+)MTZ and *R*(−)MTZ.

With regard to RTT, MTZ was able to rescue behavioral, physiological, and neuro‐morphological phenotypes in *Mecp2*‐KO male mice (Bittolo et al. 2016). The rescue of neuronal atrophy by MTZ was confirmed in an in vitro model of RTT (Nerli et al. 2020). Furthermore, in a retrospective clinical study, long‐term treatment of adult female RTT patients with MTZ was well tolerated and ensured a protection against disease progression, improving motor, sensory, and behavioral symptoms (Flores Gutiérrez et al. 2020). These effects were mirrored in adult *Mecp2* heterozygous female mice, particularly at the motor level (Flores Gutiérrez et al. 2020) and in young *Mecp2* heterozygous female mice (6 weeks old), with significant improvements in body weight, hindlimb clasping, and motor learning (Flores Gutiérrez et al. 2022). Although these preclinical and clinical studies report improvement in RTT symptoms after treatment with MTZ, its mechanism of action in RTT remains still unclear.

A major challenge in understanding the mechanism of action of MTZ is that it is a typical example of a drug with a polypharmacological profile. Indeed, the available literature reports that it can bind to 18 different G‐protein coupled receptors (GPCRs), that is, H1, 5HT2A, 5HT2C, 5HT2B, and alpha‐1A receptors, which are Gq/11‐bound, as well as 5HT7, beta‐1, beta‐2 receptors, which are Gs‐bound, and, finally, alpha‐2A, H3, kappa Opioid, LPA1, 5HT1A, alpha‐2C, D1, D2, and D3, which are Gi/o‐bound (Anttila and Leinonen 2001). However, the binding affinity of MTZ for these receptors can vary widely, and its effective binding to serotonergic receptors has been seriously questioned (Gillman 2006). We therefore hypothesized that MTZ might have additional binding partners that could explain its protective effect on RTT neurons and its ability to rescue the morphological integrity of neurons in an in vitro model of the disease that we have developed for phenotypic drug screening (Nerli et al. 2020). To test this hypothesis, we took advantage of an in silico target deconvolution approach.

The term “target deconvolution” refers to a strategy to determine a posteriori the target(s) of a drug that was effective in a phenotypic assay, that is, in a drug screen performed without any bias or presumption of drug targets (Terstappen et al. 2007; Kubota et al. 2019). Importantly, the target deconvolution process aims to demonstrate that the modulation of the targets is associated with the functional effects identified in the phenotypic assay, thereby contributing to the discovery of new mechanisms of action of the drug of interest (Terstappen et al. 2007). Today, target deconvolution methods can take advantage of a vast amount of published chemical proteomics data as well as structural information (Trosset and Cavé 2019), often related to drug discovery and development projects (Ng et al. 2016). Extensive public databases, such as ChEMBL and PubChem, contain information on drug‐protein interactions and can be interrogated by appropriate software to identify putative drug targets (Awale and Reymond 2017; Li et al. 2010; Gaulton et al. 2017) and provide the source for machine learning ligand‐based approaches (Mervin et al. 2015, 2018). However, such approaches are biased by the existence of previous studies and are therefore unlikely to provide novel targets.

In this study, as a part of a structure‐based approach, we took advantage of a large number of experimentally determined human protein crystal structures, available in the open‐access database RCSB Protein Data Bank (PDB) and preprocessed in the BioGPS software (Siragusa et al. 2015), that converts GRID Molecular Interactions Fields (MIFs) into pharmacophoric fingerprints to numerical assessment of ligand‐pocket affinity. Starting from more than 210 K crystal structures of human proteins (data accessed 10‐10‐2023) (Berman et al. 2000; Carpenter and Altman 2024), we considered only proteins that engage endogenous or exogenous “small molecules” through polar and hydrophobic interactions in specific binding pockets (about 25 K), and performed a virtual target fishing to identify the protein pockets with relevant affinity score for at least one of the two mirtazapine enantiomers, *S*(+)MTZ and *R*(−)MTZ. We provide details on how to handle the resulting list of identified protein targets putatively associated with the drug of interest in further actions such as pathway enrichment analysis, as settings and thresholds may affect the results.

## 2 Materials and Methods

### 2.1 BioGPS Screening

The BioGPS methodology, previously successfully employed for polypharmacology and target identification (Siragusa et al. 2015; Duran‐Frigola et al. 2017), is built upon the software FLAP (Baroni et al. 2007), which employs a “Common Reference Framework” to enable comparison between ligands and proteins. A wide‐range of FLAP applications include ligand‐ and structure‐based virtual screening, pharmacophore modeling and 3D‐QSAR (Tondi et al. 2016; Spyrakis et al. 2015; Cross et al. 2010, 2012; Carosati et al. 2010; Costantino et al. 2022), whereas the BioGPS approach, based on the same engine of matching quadruplets of pharmacophore points, focuses on a fast comparison of libraries of ligands and protein binding sites for applications like pocket comparison or target fishing (Lo Piparo 2019; Siragusa et al. 2016; Ferrario et al. 2014).

The used workflow encompasses the following key components: (1) defining the library of proteins, detecting potential cavities, and characterizing them by means of GRID Molecular Interaction Fields; (2) defining a library of ligands and characterizing them with the same metrics; (3) applying normalization procedures based on both binding pockets and ligands; (4) integrating the normalized scores with bioinformatics resources by creating knowledge networks linking binding pockets from PDB entries to Uniprot codes, gene symbols, and biological pathways. This integrated approach enables, for each molecule of interest, a systematic analysis of protein cavities, and the results from the following data mining for scores comparison offer valuable applications in drug discovery and molecular biology.

We included in the preliminary analysis more than 193 K pockets, that is, those protein structures available in the PDB database by filtering by organism (human); these are pre‐treated through a workflow available within the BioGPS software which includes: (i) a preliminary algorithm (named Fixpdb) which retains protein residues while eliminating water molecules, ions, and ligands; (ii) pocket identification; (iii) pocket characterization, by means of GRID Molecular Interaction Fields; (iv) data storage as a proprietary database (provided by Molecular Discovery Ltd). However, to reduce the calculation time, we finally included in the analysis only those pockets which originally contain a ligand, and further refined by limiting to structures with resolution below 2.5 Å. Thus, 25 717 pockets constituted the reference database, which will be referred to as Human‐Liganded‐Pocketome (we define the Pocketome as: “the library of about 25K pockets from human PDB entries with at least one ligand co‐crystallized within”).

Although there are several cases where details such as ions or water molecules are determinant for the establishment of ligand‐target interaction, the mentioned fixing procedure applied by the BioGPS method is justified by the screening‐like modality, which must be seen as a “fast‐but‐rough” preliminary seek for potential ligand‐pocket interactions. When applied to pocket‐pocket comparison, through the use of a large library of pockets, this approach allows tasks such as clustering of similar binding sites. Another common task is the ligand‐protein screening, with a single ligand screened versus a large library of pockets. In the current study, the pocket‐ligand comparison was extended, and the calculation involved 12 ligands (both enantiomers of MTZ and 10 additional drugs, as detailed below), versus the Pocketome.

Molecular structures of all the tested drugs were downloaded from PubChem (Kim et al. 2023) as SDF files, using a custom Python script which checked the special case of salts (including hydrochloride, maleates, and so on), in order to keep only the “parent” chemical structures. All SDF structures were annotated with their PubChem ID and phenotype characterization and merged into one single SDF file for the creation of the ligand database.

Mirtazapine as ligand was present with both *R*(−) and *S*(+) enantiomers, using the protonation option “most likely at pH 7.4” and the H, CRY, O and N1 GRID probes (https://www.moldiscovery.com/software/grid/). The grid resolution was set to 0.75 Å and the maximum number of conformations was set to 25, according to default options of the BioGPS software. Several actions were run by using commands from the command line version (CLI) of BioGPS, as detailed in Table 1.

**TABLE 1:** Command line and notes. Commands for creation of the database of ligands, the database of pockets, and the screening of all the ligands versus all the pockets.

Task	Command line	Notes
Creation of ligands database	flapdb ‐s LIGANDS.SDF ‐tag PUBCHEM_CID ‐d OUTPUT_DIR ‐ph 7.4 ‐p 4 H O N1 CRY	“‐s” is the path for the SDF files containing the structure of each of the 12 drugs; “‐tag” forces the software to use as output name the univocal ID for each drug; “‐d” is the output directory; “‐ph” sets the protonation at pH 7.4; “‐p” sets the probes to H O N1 and CRY.
Creation of a list of indices to access the pockets database	flapdbp ‐d BioGPS_DB ‐ex PocketHuman.lst	“‐d” is the path for the database of pockets; “‐ex” is the path for a file which lists the wanted pockets. This creates a file with “txt” extension, containing univocal codes for each pocket, to be used to extract the proper data from the database of pockets.
Calculation of affinity between ligands and pockets	flapvs ‐cpu 1 ‐d LigandDB ‐gb ‐4p ‐fast ‐nogs ‐noxp ‐w BioGPS_output BioGPS_DB PocketHuman.lst	“‐cpu” specifies how many CPU; “‐d” specifies the path for the database of ligands; “‐gb” specifies that Molecular Interaction Fields (MIF) are calculated over both ligands and pockets; “‐4p” sets the FLAP engine to operate by means of quadruplets of pharmacophoric points; “‐fast” specifies the speed/accuracy; “‐nogs” specifies to output only tautomer/protomer with best GlobSum value; “‐noxp” specifies to not save .xplor files; “‐w” specifies the path for the output file. The last two arguments are the database of ligands (BioGPS_DB) and the list of pockets (PocketHuman.lst).

### 2.2 Data Mining to Define ZZscores

The FLAP/BioGPS GlobSum score, representing the relative affinity of a ligand‐pocket pair, was extracted using a custom R script and saved in a matrix for the subsequent downstream analysis.

Inspired by the work of Kim and coauthors (Kim et al. 2019), the key concept that prompted us to apply a “double normalization” is that screenings focused on large comparisons along both ligands and pockets might be affected by differences in pocket and ligand size, shape, and conformations. Vice versa, a classical ligand‐vs‐pockets screening is less affected by this bias (i.e., when scores are calculated for a single ligand against a given subset of pockets), as reported in previous uses of BioGPS from literature (Duran‐Frigola et al. 2017; Lo Piparo 2019).

To tackle this problem and prioritize the best pocket candidates for each enantiomer, the GlobSum score of each ligand‐pocket combination was normalized against a background composed of the distribution of the GlobSum score of the 10 “inactive drugs”, which were experimentally determined to have no activity. In other words, with this normalization we can prioritize pockets with higher affinity for the *R*(−)MTZ and *S*(+)MTZ compared to the background of 10 “inactive drugs”. In the current study, the benchmark dataset included the following 10 “inactive” drugs: alprenolol, amiloride, felodipine, galantamine, meglumine, piroxicam, pravadoline, spectinomycin, tizanidine, and tubocurarine.

In this scenario, the use of “inactive” drugs for normalization allows the prioritization of target proteins in the biological context under study by identifying putative proteins associated with the rescue of the RTT phenotype observed in the in vitro model under study. This new score, for each ligand‐pocket interaction, was defined as a Zscore_poc using the following formula (for the pair molecule *m* and pocket *p*):

$$\begin{array}{c} \text{Zscore}\_{\mathrm{poc}}_{m,p}=\frac{\left[{\text{GlobSum}}_{m,p}–\text{mean}\left({\text{GlobSum}}_{\text{inactive drugs}}\right)\right]}{sd\left({\text{GlobSum}}_{\text{inactive drugs}}\right)} \end{array}$$

With GlobSum_inactive drugs_ being the set of GlobSum values obtained for the inactive drugs on the pocket *p*. This new score represents a statistical measurement that describes the GlobSum's relationship to the mean of GlobSum values observed for the benchmark dataset, being measured in terms of standard deviations from the mean. Thus, a Zscore of 1.0 would indicate a value that is one standard deviation above the mean.

Similarly, for a given molecule‐pocket pair, a normalized score can be obtained by considering as a benchmark all the pockets used in the calculation (roughly 25 K), with the following formula (for the pair molecule *m* and pocket *p*):

$$\begin{array}{c} \text{Zscore}\_{\mathrm{mol}}_{m,p}=\frac{\left[{\text{GlobSum}}_{m,p}–\text{mean}\left({\text{GlobSum}}_{\text{pockets}}\right)\right]}{\mathrm{sd}\left({\text{GlobSum}}_{\text{pockets}}\right)} \end{array}$$

With GlobSum_pockets_ being the set of GlobSum values obtained for the given drug on all the pockets of the Pocketome. In order to have a final value comparable for the whole dataset of drugs and pockets, these two scores are combined into the new ZZscore, which is the sum of Zscore_poc and Zscore_mol and allows to directly compare and rank different pockets, on the basis on their relative affinity with the analyzed drugs, and simultaneously compare drugs:

$${\text{ZZscore}}_{m,p}=\text{Zscore}\_{\mathrm{mol}}_{m,p}+\text{Zscore}\_{\mathrm{poc}}_{m,p}$$

For a given mirtazapine molecule, pockets with higher scores are expected to have higher affinity for such molecule compared to inactive drugs, providing tools to enhance the chance to identify biologically relevant drug‐target interactions for the studied pathology. In the present study, these scores were used comparatively along the Pocketome to identify biologically relevant pocket‐molecule pairs, to be further investigated by dedicated literature searches and guide further experiments. An overall threshold of 2 was used for ZZscore to filter out irrelevant pocket‐molecule pairs, but in some cases additional but less strict filters were based on the two addends, Zscore_poc and Zscore_mol, and the threshold of 0.8 (for each addend) was selected as default value for the analysis.

### 2.3 Bioinformatics Analysis

After retrieving gene names from the given list of relevant pockets, data for gene‐pathway relationships were extracted from Reactome (Fabregat et al. 2018), a well‐known source for pathways and gene‐pathway relationships. To facilitate data storage and analysis, an SQLite database was created, including the information of pockets, classification of molecules, and the calculated scores for original GlobSum, Zscore_mol, Zscore_poc, and ZZscore values. gene set enrichment analysis (GSEA) was carried out by means of the “gost” function of the R package *gprofiler2*, with the following parameters (default values): exclude_iea = True; user_threshold = 0.05; ordered_query = True; significant = True; evcodes = True; correction_method = “fdr”. We identified the candidate pockets for each drug, which were manually inspected using the BioGPS software (with the graphical user interface) to confirm their possible interaction with the corresponding pocket(s).

To investigate the potential enrichment of RTT‐associated genes among the prioritized protein targets identified for both MRT enantiomers, an additional GSEA preranked analysis was performed (Subramanian et al. 2005). Rett‐associated gene targets were retrieved from the Open Targets Platform (OpenTargets), which aggregates multiple public data sources and enables researchers to identify and prioritize potential therapeutic drug targets by means of a set of scores for target‐disease associations (Ghoussaini et al. 2021). This list was filtered to retain only those targets annotated to contain at least one ligand and one pocket. Such targets, ranked by decreasing disease‐target association scores, constituted an artificial pathway of Rett‐associated genes. For each enantiomer, the input ranked list for GSEA analysis was generated by sorting all tested proteins based on their decreasing values of ZZscore. The potential enrichment of Rett‐associated targets was assessed using the GSEA pre‐ranked test from the clusterprofiler R package, and statistical significance was assessed with 10 K permutation tests by considering significant results with an adjusted *p*‐value < 0.05 and a normalized enrichment score (NES) > 0 (Yu et al. 2012). The enrichment score (ES) quantifies the extent to which a protein set is overrepresented either at the top or bottom of a ranked list. To account for variations in set size, the ES is normalized, resulting in the normalized enrichment score (NES). The use of NES facilitates the comparison of analysis results across different sets and is calculated by dividing the ES by the mean ES obtained from all permutations of the dataset.

### 2.4 Mouse Genotyping

The animal use was approved by the Italian Ministry of Health, in accordance with the Italian legislation D.Lgs 116/92 with authorization n. 693/2021‐PR issued on 07‐09‐2021, having E.T. as authorized principal investigator. The animals were housed under standard conditions in ventilated cages, maintained on a 12‐h light/dark cycle, with food and water available ad libitum. The C57BL/6 mice were purchased from Charles River Laboratories (Calco, LC, Italy); the Wild‐type (WT) male mice were crossed with female heterozygous in Mecp2 gene (Guy et al. 2001) (*Mecp2* ^*−/y*^, B6.129P2(C)‐Mecp2tm1.1Bird/J, stock: 003890, Jackson Laboratories, Bar Harbor, Maine; RRID:IMSR_JAX:003890) in order to obtain WT (*Mecp2* ^*+/y*^, WT) and Knock‐Out (*Mecp2* ^*−/y*^, KO) male mice. Mice genotyping was performed at postnatal day 0 (P0), by extraction of the DNA from the dissected tails. Pup tails were previously anesthetized with a frozen ice‐cube (normally kept at −20°C) and then a small part of the tails was cut and put in 500 μL tubes. Thus, mice are kept in the oven in single boxes (at 37°C) and each box and small tube was signed with a corresponding number, in order to be sure to link each genotype to the right mouse. The DNA extraction was performed by KAPA Express Extract Buffer and KAPA Express Extract Enzyme in line with the instructions. The Polymerase Chain Reaction (PCR) was performed using KAPA2G Fast DNA polymerase with the following primers:

Common forward (9875, Jackson Laboratory): 5′AAATTGGGTTACACCGCTGA‐3′.

Wild‐Type Reverse (oIMR7172, Jackson Laboratory): 5′‐CTGTATCCTTGGGTCAAGCTG −3′.

Mutant Reverse (9877, Jackson Laboratory): 5′‐CCACCTAGCCTGCCTGTACT‐3′

For each genotyping, water was used as negative control and heterozygous DNA from female mice as positive control. The final volume for the PCR reaction was 25 μL and the program settings were: 3 min of initial denaturation at 95°C followed by 20 s of denaturation at 95°C, annealing for 20 s, elongation 72°C for 20 s (35 cycles) and final elongation at 72°C for 2 min. PCR products were separated on a 1.5% agarose gel using 0.004% of Biotium (Gel red) and a single amplified band of 465 base pairs was considered for the WT allele, a band of 240 base pairs for the KO and both bands for the positive control (heterozygous female). We performed genotyping before mice suppression, in order to minimize the number of animals and reagents per experiment. Overall, we used 10 mice (5 for WT and 6 for KO).

### 2.5 Primary Hippocampal Cultures of WT and *Mecp2* ^*−/y*^ Neurons

Primary hippocampal neuronal cultures were prepared from P0 male mice, both WT (*Mecp2* ^*+/y*^) and KO (*Mecp2* ^*−/y*^), as previously described (Guy et al. 2001). Mice were sacrificed by decapitation, and the hippocampi were then carefully extracted from the brain, under a bright microscope, and put in cold Hank's balanced salt solution (HBSS) (NaHCO_3_ 4.2 mM, Hank's salt powder 0.952%, HEPES 12 mM, 4‐(2‐hydroxyethyl)‐1‐piperazineethane‐sulphonic acid, Sigma, REF. H2387). The tissue dissection was achieved by adding 0.25% Trypsin (Euroclone, ref. ECB3051D) for 8 min at 37°C. The enzymatic digestion was blocked with Dulbecco's Modified Eagle Medium high glucose (DMEM, Invitrogen, ref. 41 965 039), supplemented with 10% fetal bovine serum (FBS, Euroclone, ref. ECS5000L) and penicillin–streptomycin (Euroclone, ref. ECB3001D). In order to obtain precipitation, the tissue was centrifugated at 800 rpm for 5 min at RT. The tissue was then resuspended with 1 mL of DMEM and mechanically triturated. Cells were counted in the Burker chamber (Eppendorf), following the dye exclusion method using Trypan blue (Sigma) and the number of cells extracted for each pup was around 700.000. Cells were plated on 96 well plates (MW) (Sarstedt, ref. 833 924) and in 24 well plates (Sarstedt, ref. 833 922) previously treated with 0.2% poly‐Ornithine (Sigma, ref. P3655‐100MG). Cells were seeded at different concentrations: 160 cell/mm^2^ in 96 well plates and 640 cell/mm^2^ in 24 Multi‐Well (MW). In average, a pair of hippocampi explanted bilaterally from a single mouse brain was split into 6 wells of a 24 MW plate and 40 wells of a 96 MW plate. Hence, *n* = 1 is referred to cultures from a single unpooled mouse brain. Once seeded, cells were put in the incubator at 37°C and 5% CO_2_ for 1 h to allow the cells to adhere; then the DMEM was substituted with Neurobasal (Invitrogen, ref. 21 103 049) supplemented with 2% B27 (Invitrogen, ref. 17 504 044), 1 mM L‐glutamine, and 1% penicillin–streptomycin. To inhibit nonneuronal cell proliferation, D‐arabinofuranoside (Ara‐C, Sigma, ref. 251 010‐1GM) was used at 2.5 μM as final concentration (cell culture medium change at 3 days in vitro DIV 3). Cells were maintained in cultures for 6 days in 96 well plates and for up to 9 days in 24 well plates. During all the procedures, WT and *Mecp2* ^*−/y*^ hippocampi were kept separate in order to have isolated cultures of both conditions.

### 2.6 Neuronal Cultures Treatments

*Mecp2* ^*−/y*^ cells seeded in 96 well‐plates were treated from DIV 3 to DIV 6 with alprenolol, amiloride, felodipine, galantamine, meglumine, piroxicam, pravadoline, spectinomycin, tizanidine, and tubocurarine at a final concentration equal to 10 μM. *Mecp2* ^*−/y*^ cells were treated with the vehicle DMSO 0.1% as the control condition. We set *N* = 1 for biological replicates, which corresponds to 1 independent cell culture from 1 mouse, which was sufficient to test all drugs at once within the same 96 MW plate, in technical duplicate. Drugs and the control solution were coded by an experimenter different from the one who carried out the analysis, who was therefore blinded to thetreatments.

### 2.7 Immunofluorescence

Hippocampal neurons seeded in 96 MW plates were fixed at DIV 6 after 72 h of treatments which were started at DIV 3. Fixation was performed with PFA 4% for 15 min and then cultures were washed with PBS 1X. Permeabilization of cell membranes was achieved using PBS‐Tryton 0.1% (PBS‐T) and 2% of Bovine Serum Albumin (BSA, Sigma). Primary antibodies (Abs) were diluted in blocking solution (PBS‐T in 2% BSA) and cells were incubated for 90 min at RT, in a dark humified chamber and on a rocker. Primary Abs used: rabbit anti‐MAP2 isotypes, (ab32454, Abcam; 1:500); mouse anti‐NeuN isotypes, (LS‐C312122‐100, LSBio; 1:500). Thereafter, cells were washed twice with PBS 1X (5 min/wash) and then incubated with secondary Abs for 90 min at RT, in a dark humified chamber and on a rocker. Secondary Abs used: anti‐rabbit IgG Alexa Fluor568 (A10042, Invitrogen); anti‐mouse IgG Alexa Fluor488 (A11001, invitrogen). Both Abs were diluted 1:1000 in blocking solution (PBS‐Tryton 0.1% in BSA 2%). Finally, cells were washed with PBS‐Tryton 0.1% and with PBS 1X (5 min/wash) and then incubated with Hoechst 33342 (10 mg/mL, Sigma, ref. B2261‐100MG) at the dilution 1:1000 (final concentration 10 μg/mL) in PBS 1X for 7 min. Hoechst was finally washed with PBS 1X.

### 2.8 Image Acquisition

Fluorescent images were captured using a Nikon Eclipse Ti‐E epifluorescence live imaging microscope, equipped with a Nikon DS‐Qi2 camera (CMOS sensor, 16.25 megapixel), and a 10X objective lens. All the acquisitions were performed using the software Nis‐Elements 4.60 with the module “JOBS” for automated imaging. Eleven small images (3.0 × 3.0 fields) per single well were acquired using the 10X objective. Image size was 14 bit‐1636 × 1088 pixels, which corresponds to 1.440 × 0.957 mm. The number of seeded neuronal and nonneuronal cells was respectively determined by automatic counting of NeuN‐positive neurons and Hoechst‐positive cells, with the “Objective‐analyzer” plugin for Nis‐Element 4.60. To obtain the best signal to perform the image analysis, image acquisition was carried out using the most performant ratio between exposure time (500 msec for MAP2, 1 s for NeuN, 10 msec for Hoechst) and filters (1, the minimum).

### 2.9 Image Analysis

The software NeuriteQuant was used to perform morphological analysis of neurons at DIV 6 (Baj et al. 2014). Morphological parameters quantified were: (i) Total dendritic length (TDL): sum of the length of the entire dendritic arborization of a neuron; (ii) Endpoints: number of terminal points counted at the end of visible dendritic staining (MAP2). To achieve the most accurate dendritic tracing, the Neurite cell body detection threshold was significantly increased. This adjustment is necessary because if the body detection is not perfectly set, dendrites located near the soma may go undetected, leading to imprecise TDL measurements.

### 2.10 Neuronal Protein Extraction and Quantification

WT and *Mecp2* ^*−/y*^ hippocampal neurons were seeded in 24 MW plates at DIV 0. At DIV 3, Ara‐C was added to the cells. At DIV 9, treatments were administered over 15 min as follows: DMSO 0.1% (control condition), mirtazapine (MTZ) 10 μM. Then, proteins were extracted and quantified. For each well, 30 μL of ice‐cold RIPA lysis buffer supplemented with HALT protease (1:100) and HALT phosphatase (1:100) inhibitors were added. Then, protein extracts were sonicated (4 cycles of 4 s with an amplitude of 10%) and centrifuged (4°C, 10′, 13 000 rpm). The supernatant was collected and quantified. The standard curve of BSA was prepared starting from a stock solution of 0.1 mg/mL (albumin standard 2 mg/mL, Thermo Fisher 23 209). The absorbance (595 nm) was read using a multi‐detection plate reader (Promega Glomax) and plotted in an excel file to obtain the regression line. To verify the quality of Bradford quantification, only regression lines with *R* ^2^ = 0.99 were considered. Experiments were carried out on *n* = 5 WT and *n* = 5 *Mecp2* ^*−/y*^ independent hippocampal neuronal cultures prepared from unpooled mice (each culture = 1 mouse brain). No blinding procedure was needed for these experiments. The sample size was empirically determined on the basis of previous lab experience.

### 2.11 Dot‐Blot Analysis

The dot blot analysis was performed in order to identify the proper protein concentration between 30 μg and 50 μg to use in the Western blotting analysis. Protein extracts were blotted onto a nitrocellulose membrane for 10 min using a dot blot rack. The membrane was stained with Ponceau Red stain to validate the blotting. The membrane was then blocked with nonfat milk in TBS‐Tween 0.1% for 45 min and then incubated overnight at 4°C with primary Abs (anti‐pERK and anti‐totalERK, 1:1000 3% BSA in TBS‐T). The day after, washes were performed (5 min × 3 times) and the membrane was incubated (1 h at RT, stirring) with secondary antibody (anti‐mouse and anti‐rabbit Horseradish peroxidase, 1:5000 5% nonfat dry milk in TBS‐T). Proteins were visualized by chemilunescent detection using WesternBrigthTM ECL reagent in ChemiDoc (Biorad 6873).

### 2.12 Western Blot Analysis

To perform Western blot analysis, 30 μg of protein extracts were first denatured for 5 min at 95°C using a mixture of 1:4 (v/v) sample/4x SDS sample buffer, and then separated in 10% SDS‐PAGE gel. The condition of the electrophoretic run was set as following: 300 V and 20 mA (per gel). Samples were transferred onto a PVDF membrane using a trans‐blot semi‐dry system (Biorad) with the following settings: 18 V, 200 mA for 1 h. The membrane was stained with Ponceau Red stain (15 min) and detected via colorimetric imaging at ChemiDoc. The membrane was then blocked with 5% nonfat milk in TBS‐Tween 0.1% (TBS‐T) solution (1 h, RT) and then incubated with primary Abs anti‐p‐ERK1/2 (mouse, M9692 Sigma) and anti‐total‐ERK1/2 (rabbit, M5670 Sigma): 1:1000 3% BSA in TBS‐T over‐night at 4°C. After three washes with TBS‐T (10 min RT), the membrane was incubated with the secondary Abs 1:1000 3% BSA in TBS‐T for 1 h at RT: anti‐mouse AlexaFluor 488 (A11001, Invitrogen) and anti‐rabbit AlexaFluor 488 (A11034, Invitrogen). Bands were then detected on ChemiDoc (Bio‐Rad) and quantified with ImageLAB software (free download at: https://www.bio‐rad.com/). The lane and band analysis was performed considering that this method has been reported to be more accurate, as it automatically subtracts the specific local background to the band of interest (“adjust total band volume”). The normalization was then performed on the adjusted value of the total protein (total ERK).

### 2.13 Statistical Analysis

All statistical analysis and data representation were conducted using Prism 8.0 software (Graphpad), while data organization was performed with Microsoft Excel (2308, build 16731.20234) (Office). All data were first tested for normality using the Shapiro–Wilk test. Since data were normally distributed, the one‐way ANOVA test was used to calculate the statistical difference when comparing more than two groups. Post hoc Dunnett's multiple comparison was performed to determine the effect of all the drugs versus control (DMSO 0.1%). Two‐way ANOVA was used for multiple comparisons between more than two groups (two variables: genotype and drug treatments). In column graphs, the mean of all measurements was plotted with the corresponding standard error (SEM). Grubbs' test was conducted to determine possible outliers. In all the experiments, no animals were excluded from the analysis.

## 3 Results

### 3.1 Combined In Vitro and In Silico Strategy for Target Identification

Most drugs exert their biological effects through protein‐ligand interactions (Carpenter and Altman 2024), and computational methods are commonly used to predict such interactions, usually by numerically comparing several ligands against the same target. However, difficulties arise when screening the same library of ligands against different targets, as the size and shape of the ligands and binding sites can affect the ranking of molecule‐pocket pairs. We present here the results of a procedure inspired by the work of Kim and coworkers (Kim et al. 2019) who applied a double normalization to the docking results across ligands and receptors.

**FIGURE 1:**
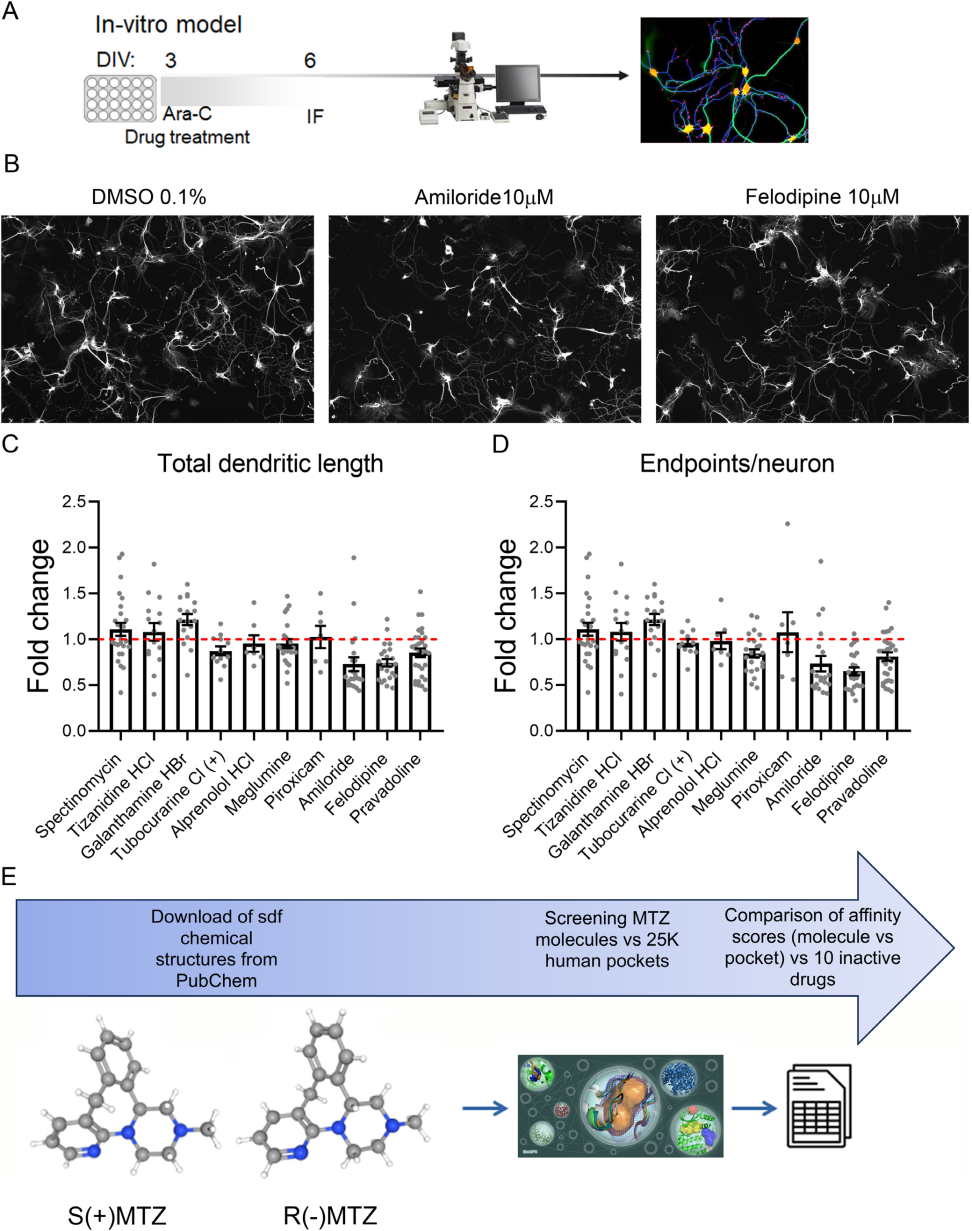
Combined in vitro and in silico workflow for inverse docking target identification. (A) In vitro model to detect drug effects on *Mecp2‐KO* (RTT) neurons. Hippocampal primary cultures were seeded at DIV 0 in 96 Multi‐Well plates. After 3 days, Ara‐C and drug treatments were administered to the cells. At DIV 6, cells were fixed and immunofluorescence was performed to label dendrites and soma. Images were acquired at the Nikon Eclipse Ti‐E epifluorescence microscope (10x magnification objective) and each image was analyzed with the NeuriteQuant software. (B) Representative images for NeuriteQuant morphological analysis of Total Dendritic Length (TDL) and Endpoints (EPs) of DIV 6 hippocampal *Mecp2*‐KO neurons, plated at the density of 160 cells mm^2^. From left, *Mecp2*‐KO neurons treated with DMSO 0.1% (control condition), Amiloride and Felodipine at the concentration of 10 μM. (C, D) Treatments of neuronal cultures with 10 inactive molecules. Quantitative data of *Mecp2*‐KO neurons, reporting (C) the average TDL per neuron (fold change) and (D) the average number of EPs per neuron. *n* = 11 images for a total of 1 independent biological replicate (cell cultures). Error bars = mean ± SEM. Red line control condition normalized to = 1 (DMSO 0.1%). One‐way ANOVA (two‐tailed) with Dunnett's multiple comparisons test vs. DMSO conditions. ***p < 0.001, **p < 0.01, *p < 0.05. Grubbs' test was conducted to identify outliers. (E) In silico prediction method. First step: Download of *R*(−)MTZ and *S*(+)MTZ chemical structures from PubChem as SDF files; second step: BioGPS screening of *R*(−)/*S*(+) MTZ versus 25 717 human pockets; third step: Comparison of affinity scores versus 10 inactive molecules. A one‐way ANOVA was performed to compare the effects of the ten drugs and the control (DMSO 0.1%). For TDL, F(10, 221) = 4.48, *p*‐value = 0.0001. For EP, F(10, 221) = 4.41, *p*‐value = 0.0001. Dunnett's multiple comparison test was then used to compare each drug to the control. Adjusted *p*‐values for the TDL condition were: Spectinomycin = 0.99, Tizanidine HCl = 0.99, Galanthamine HBr = 0.58, Tubocuranine Cl (+) = 0.98, Alprenolol HCl = 0.99, Meglumine = 0.99, Piroxicam = 0.99, Amilopine = 0.54, Felodipine = 0.58, Pravadoline = 0.98. Adjusted *p*‐values for the EP condition were: Spectinomycin = 0.99, Tizanidine HCl = 0.99, Galanthamine HBr = 0.74, Tubocuranine Cl (+) = 0.99, Alprenolol HCl = 0.99, Meglumine = 0.92, Piroxicam = 0.99, Amilopine = 0.38, Felodipine = 0.077, Pravadoline = 0.63.

To investigate the mechanisms of action of *R*(−)MTZ and *S*(+)MTZ enantiomers, we combined in vitro and in silico methods to identify possible novel protein targets and associated pathways through an “inverse docking” or “target fishing” approach. To reduce the probability of fishing false positive targets, we decided to filter our in silico analysis against a baseline “noise” of 10 structurally different inactive drugs. We define inactive drugs as molecules that had no effect on two morphological parameters, that is, the total dendritic length (TDL) and the number of dendritic endpoints/neuron (EP), that define neuronal atrophy in an in vitro model of RTT developed for phenotypic drug screening (Nerli et al. 2020) (Figure 1A). These drugs, for which we show the lack of efficacy on both total TDL (Figure 1B) and EP (Figure 1C), are: alprenolol, amiloride, felodipine, galanthamine, meglumine, piroxicam, pravadoline, spectinomycin, tizanidine, tubocurarine.

The use of inactive drugs as baseline normalization allows the prioritization of *R*(−)MTZ and *S*(+)MTZ target proteins in the specific biological context under study with the goal to restrict the analysis to the target proteins associated with the rescue of the RTT phenotype observed in the in vitro model. Then, we calculated the GlobSum score of both MTZ enantiomers and of the 10 inactive drugs against the human pocketome. We used the BioGPS software to calculate the affinity against a pocket database consisting of 25 717 human pockets (Figure 1D). These pockets were selected from the Protein Data Bank (PDB) based on three requirements, that is, to be: (a) from a human protein with available 3D crystal structure; (b) with resolution below 2.5 Å; and (c) bound by a ligand (see methods).

The GlobSum affinity score includes the contribution of all the GRID probes: H, for shape comparison between ligand and pocket; N1 and O, for matching polar groups, with complementary hydrogen bonding character; and CRY, for matching regions with hydrophobic character. For each *R*(−)MTZ or *S*(+)MTZ‐pocket interaction, the GlobSum score was normalized twice: we named Zscore_mol the normalization against a background (mean and standard deviation) obtained for the same molecule over the whole set of pockets, and Zscore_poc the normalization (for each pocket) against the background given by the 10 inactive drugs. The obtained ZZscore (which is the sum of Zscore_poc and Zscore_mol) was used to rank the molecule‐pocket pairs; the whole procedure was performed for both the MTZ enantiomers.

**FIGURE 2:**
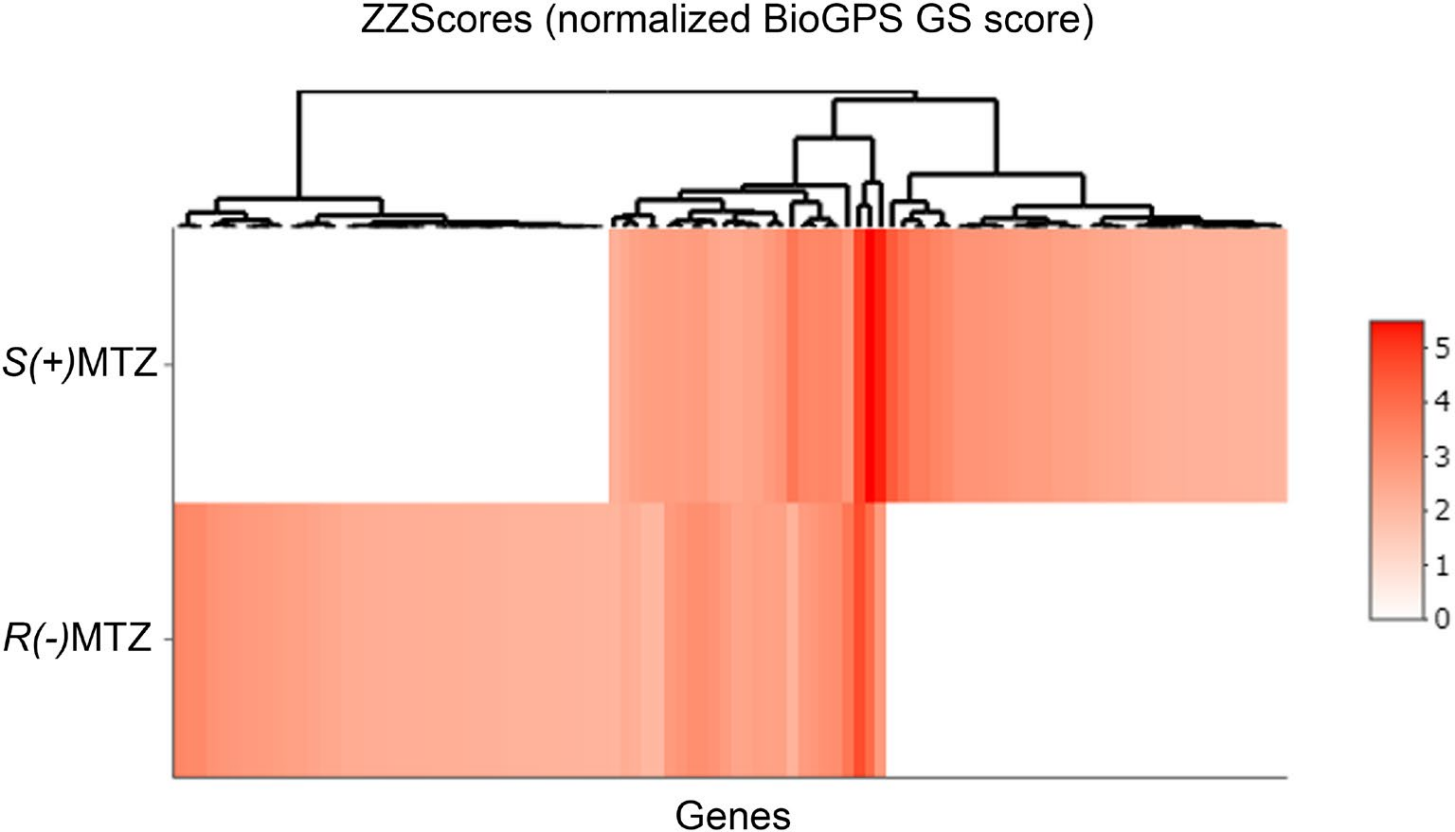
Heatmap of *S*(+)MTZ and *R*(−)MTZ pocket affinity. The heatmap shows the target genes for *R*(−) and/or *S*(+) mirtazapine (according to their pockets), filtered by using ZZscore values ≥ 2.0, Zscore_poc ≥ 0.8 and Zscore_mol ≥ 0.8. The cluster dendrogram is reported on the top of the plot: Dark red refers to genes with highest affinity, light red to genes with lowest affinity.

**FIGURE 3:**
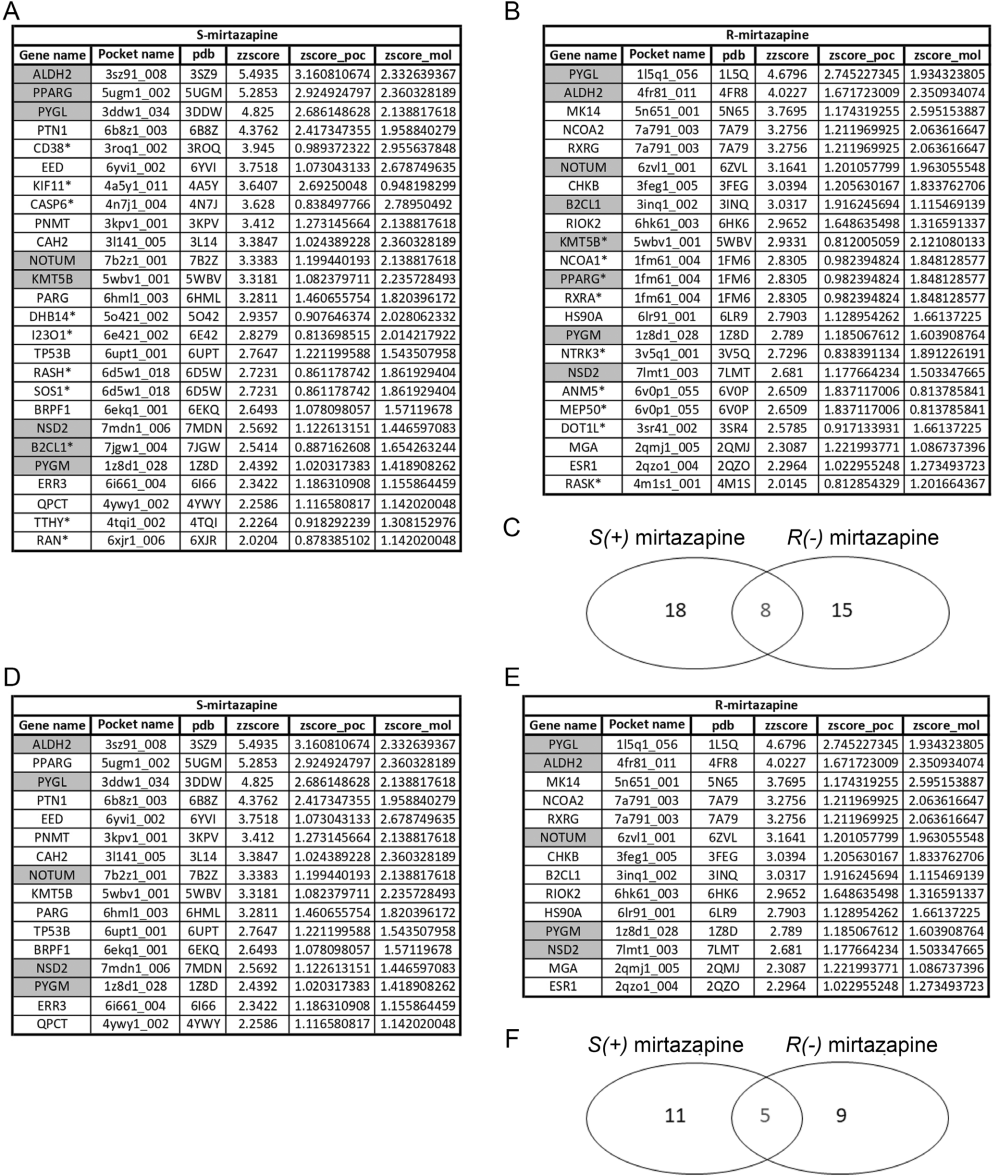
In silico predicted target genes for *R*(−)MTZ and *S*(+)MTZ. List of (A) 26 target genes for *S*(+)MTZ and (B) 23 target genes for *R*(−)MTZ predicted by the procedure when setting thresholds as follows: ZZscore ≥ 2.0, Zscore_poc ≥ 0.8 and Zscore_mol ≥ 0.8. In gray: Common genes. * = genes which are lost in tables D and E. (C) Venn diagram showing 8 genes in common between table A and table B. List of (D) 16 target genes for *S*(+)MTZ and (E) 14 target genes for *R*(−)MTZ predicted by ZZscore ≥ 2.0, Zscore_poc ≥ 1.0 and Zscore_mol ≥ 1.0. In gray: Common genes. (F) Venn diagram showing 5 genes in common between table D and table E.

Figure 2 reports a heatmap with the ZZscore values ≥ 2.0 of the target genes for *R*(−) and/or *S*(+) mirtazapine (according to their pockets). The heatmap highlights the presence of common target genes for *R*(−)MTZ and *S*(+)MTZ. To set the appropriate cut‐off parameters to select the targets with the highest predicted affinity, the list of the retrieved proteins with the ZZscore values ≥ 2.0 was further filtered according to the cut‐off parameters Zscore_poc ≥ 0.8 and Zscore_mol ≥ 0.8 (Figure 3A,B).

While Kim et al. (2019) assigned different weights to the two terms (70% and 30% to the Zscore over the receptor and over the ligand, respectively) but without using any thresholds, we decided to give the same importance to the two scores (i.e., we summed them). Furthermore, we applied filters in order to keep all the cases with excellent molecule‐pocket affinity (by setting to 2.0 the threshold for ZZscore) while excluding cases in which the affinity is due only to one of the two terms (the additional filters for single Zscore were set to 0.8). With these cut‐off parameters, the prediction of protein targets made by the algorithm was reduced to 26 targets for *S*(+)MTZ (Figure 3A) and 23 for *R*(−)MTZ (Figure 3B). Notably, the different enantiomers were predicted to bind to different pockets related to the same gene (e.g., for ALDH2 *S*(+)MTZ binds to the PDB entry 3SZ9 whereas *R*(−)MTZ binds to the PDB entry 4FR8). Interestingly, only in two cases did the same pocket have good affinity values for both enantiomers: pocket 001 of the PDB entry 5WBV (corresponding to the gene KMT5B) and pocket 028 of the PDB entry 1Z8D (corresponding to the gene PYGM). However, looking at genes instead of pockets or even the PDB entries, 8 genes were found to be common between the lists obtained for the two enantiomers (in gray, Figure 3A–C).

To further investigate the effect of the used threshold on the results, we filtered the same protein lists by using more stringent parameters, that is, ZZscore ≥ 2.0, Zscore_poc ≥ 1.0, and Zscore_mol ≥ 1.0 (Figure 3D,E). Using this more restrictive setting, we retrieved 16 target proteins for *S*(+)MTZ and 14 for *R*(−)MTZ, with 5 proteins in common (in gray; Figure 3D–F). Thus, when the more stringent filters were applied, a total of 10 target proteins were lost for *S*(+)MTZ and 9 for *R*(−)MTZ, three of which were common to both enantiomers. In conclusion, employing the most stringent conditions, *S*(+)MTZ was predicted to bind to 16 novel protein targets, namely: **ALDH2**, PPARG, **PYGL**, PTN1, EED, PNMT, CAH2, **NOTUM**, KMT5B, PARG, TP53B, BRPF1, **NSD2**, **PYGM**, ERR3, and QPCT. Conversely, *R*(+)MTZ was predicted to bind to 14 novel protein targets. These include **PYGL**, **ALDH2**, MK14, NCOA2, RXRG, **NOTUM**, CHKB, B2CL1, RIOK2, HS90A, **PYGM**, **NSD2**, MGA, and ESR1 (see list of abbreviations for full gene names). Five of these genes are shared between *S*(+)MTZ and *R*(+)MTZ, namely: ALDH2, NOTUM, NSD2, PYGL, and PYGM (highlighted in bold in the aforementioned lists).

### 3.2 Experimental Test for Putative False Positives

The loss of a relatively large number of potential targets when using more stringent parameters raised the question of whether the use of more relaxed parameters could generate false positives. To gain more insight into this hypothesis, we focused on those targets lost when applying the more stringent filtering, that is, when using 1.0 instead of 0.8 as the threshold for the Zscores. Numerically, the threshold of 1.0 means that the GlobSum scores of the selected molecule–pocket candidates should exceed their corresponding mean values (along molecules or pockets) by at least the value of one standard deviation.

In particular, during the visual inspection of the pockets (and corresponding genes) that were lost when increasing the thresholds, we were intrigued by the complex of Son of sevenless homolog 1 (SOS1) and the GTPase Harvey Rat sarcoma virus (RASH), identified as a possible target for both *S*(+)MTZ (with ZZscore = 2.72) and *R*(−)MTZ (with ZZscore = 2.02). On the one hand, the modulation of such a complex may have a biological significance, as discussed below. On the other hand, the pocket identified by the software (pocket 018 of the PDB entry 6D5W) had a peculiar shape, as it appeared very large and complex compared to most of the other pockets, which are more globular. Scrolling through the lists of pockets for the same gene, the second PDB entry related to the same gene was 6CUO, with pocket 006 having a more globular shape but worse ZZscore values, with only *R*(−)MTZ slightly above the threshold (2.01 i.e., the same magnitude of what observed for the PDB entry 6D5W), while for *S*(+)MTZ we obtained a negative value.

**FIGURE 4:**
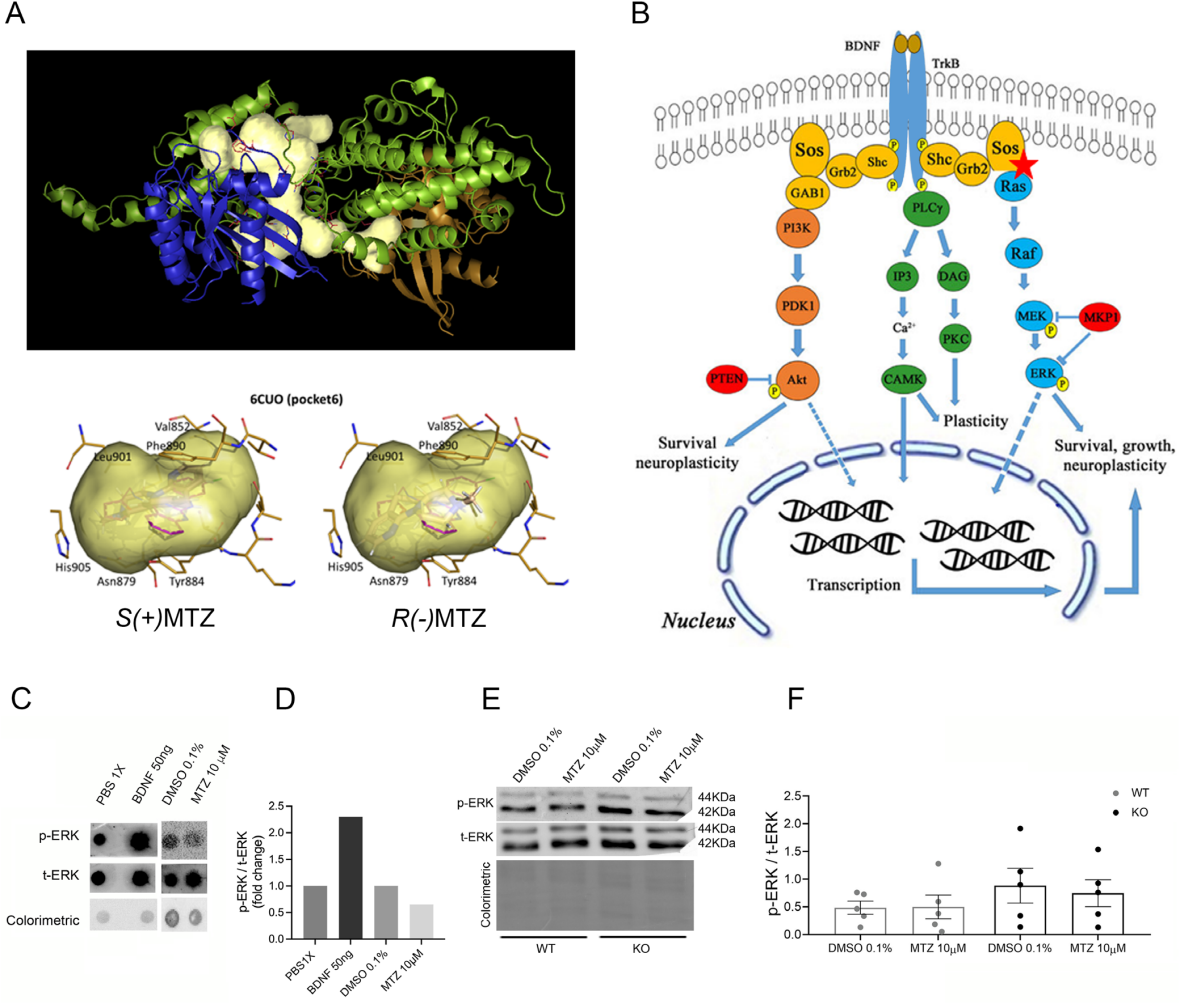
BioGPS analysis showed high affinity of MTZ to SOS1/RASH protein complex. (A) Top: 3D reconstruction of the HRAS/SOS1 complex, with pocket 6d5w1_018. Green = chain S (PDB entry = 6D5W, protein name = Son Of Sevenless homolog1, SOS1); blue = chain R (PDB entry = 6D5W, protein name = GTPase HRas, HRAS); gold = chain Q (PDB entry = 6D5W, protein name = GTPase HRas, HRAS); yellow: Pocket = 6d5w1_018, PDB entry = 6D5W. Bottom: Pocket of another PDB entry (6CUO) for the same target, with the two enantiomers docked within, with worst values of ZZscores. (B) Hypothesis of the mechanism of action (MoA) of MTZ by activation of the Ras/MAPK signaling pathway through direct binding to a pocket of the SOS1/RASH protein complex (red cross) (Dehmelt et al. 2011). (C, D) Dot‐blot (*n* = 1), and (E, F) Western‐blot of p‐ERK1/2 (ser 42–44) at DIV 9 after 15 min of treatment with DMSO 0.1% and mirtazapine (MTZ) 10 μM (*n* = 5 independent cell cultures; error bars = mean ± SEM). A two‐way ANOVA was performed to evaluate the main effect of genotype and treatment. No significant interaction between genotype and treatment was detected F (1, 4) = 1.105, *p*‐value = 0.35, as well as genotype (F (1, 4) = 1.442, *p*‐value = 0.30) or treatment effects (F (1, 4) = 0.576, *p*‐value = 0.49). Grubbs' test was conducted to identify outliers.

Such a protein complex is biologically relevant as it represents a key step in the signaling cascade activated by several trophic and growth factors and is occupied by specific activators. In particular, pocket 006 of the PDB entry 6CUO can be bound by the ligand FFS (*N* ~ 2 ~ −(3‐chlorophenyl)‐*N* ~ 4 ~ −[(furan‐2‐yl)methyl]quinazoline‐2,4‐diamine) (Alam et al. 2016), while the large pocket 018 of the PDB entry 6D5W is located at the interface between SOS1 and RASH (Figure 4A) and contains the ligand GNP (phosphoaminophosphonic acid‐guanylate ester). Another pocket (004) of the same PDB entry contains another activator, FVV (10‐[(4‐fluorophenyl)methyl]‐2,3,4,10‐tetrahydropyrimido[1,2‐a]benzimidazole) (Abbott et al. 2018) but ZZscore values are close to 0 for both enantiomers.

In the nervous system, SOS1 is critical for activation of the extracellular signal‐regulated kinases (ERK) signaling cascade (Figure 4B, in blue), downstream activation of TrkB, the receptor for the neurotrophin brain‐derived neurotrophic factor (BDNF) (Hodges et al. 2018; Kumar et al. 2005; Meng et al. 2005). To investigate the involvement of these targets, Western‐blotting analysis for protein levels of the ratio of phosphorylated ERK (p‐ERK)/total ERK (t‐ERK) was performed on primary cultures of WT and RTT (*Mecp2* ^*−/y*^, indicated as KO) hippocampal neurons at day in vitro (DIV) 9 treated for 15 min with MTZ 10 μM or the vehicle solution DMSO 0.1% (control condition). Optimization of the experimental conditions for protein concentration (30 μg) and antibody dilution (1:1000) was determined by dot blot analysis, which also established the optimal treatment time (15 min), based on the phosphorylation of ERK induced by the incubation of the neuronal cultures with BDNF 50 ng/mL or MTZ 10 μM (Figure 4C,D). Western‐blotting analysis revealed a not significant increase in ERK phosphorylation in KO compared to WT even when treated with vehicle alone (DMSO 0.1%) (Figure 4E,F). Thus, these data suggest that in order to minimize the chance of having false positive targets, as in the case of SOS1 and RASH, it is crucial: (i) to check the shape of the pocket, removing cases of irregular shape; (ii) to use more stringent thresholds. Accordingly, in the subsequent enrichment analyses we used the aforementioned set of more restrictive parameters and carefully checked the shape of the pockets, as described in the next section.

### 3.3 Inverse Docking for Target Identification

**FIGURE 5:**
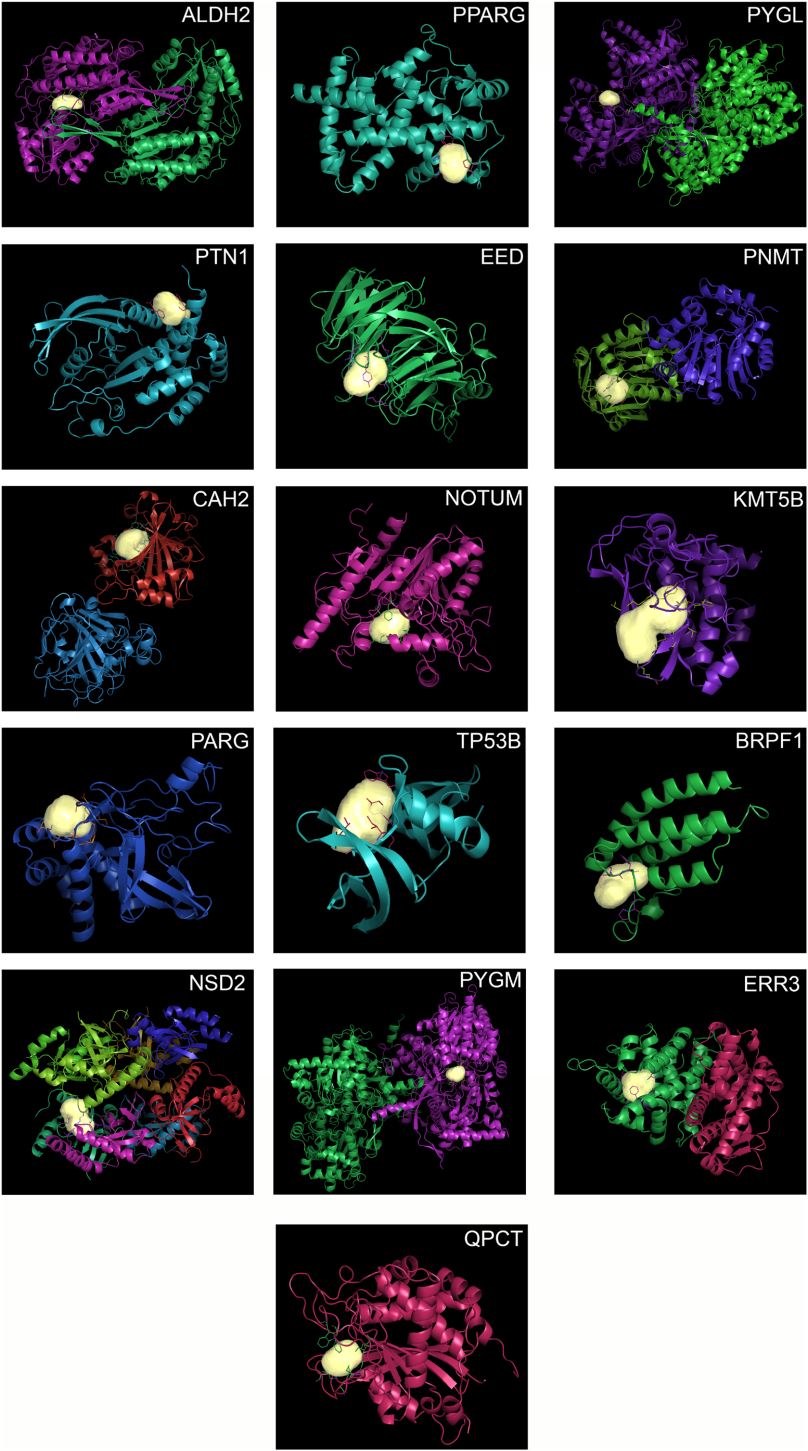
The 16 proteins with binding pockets with high affinity for *S*(+)MTZ. 2D images of 3D reconstructed protein complexes resulted in having high binding affinity with *S*(+)MTZ. 3D reconstructions were performed on BioGPS software.

**FIGURE 6:**
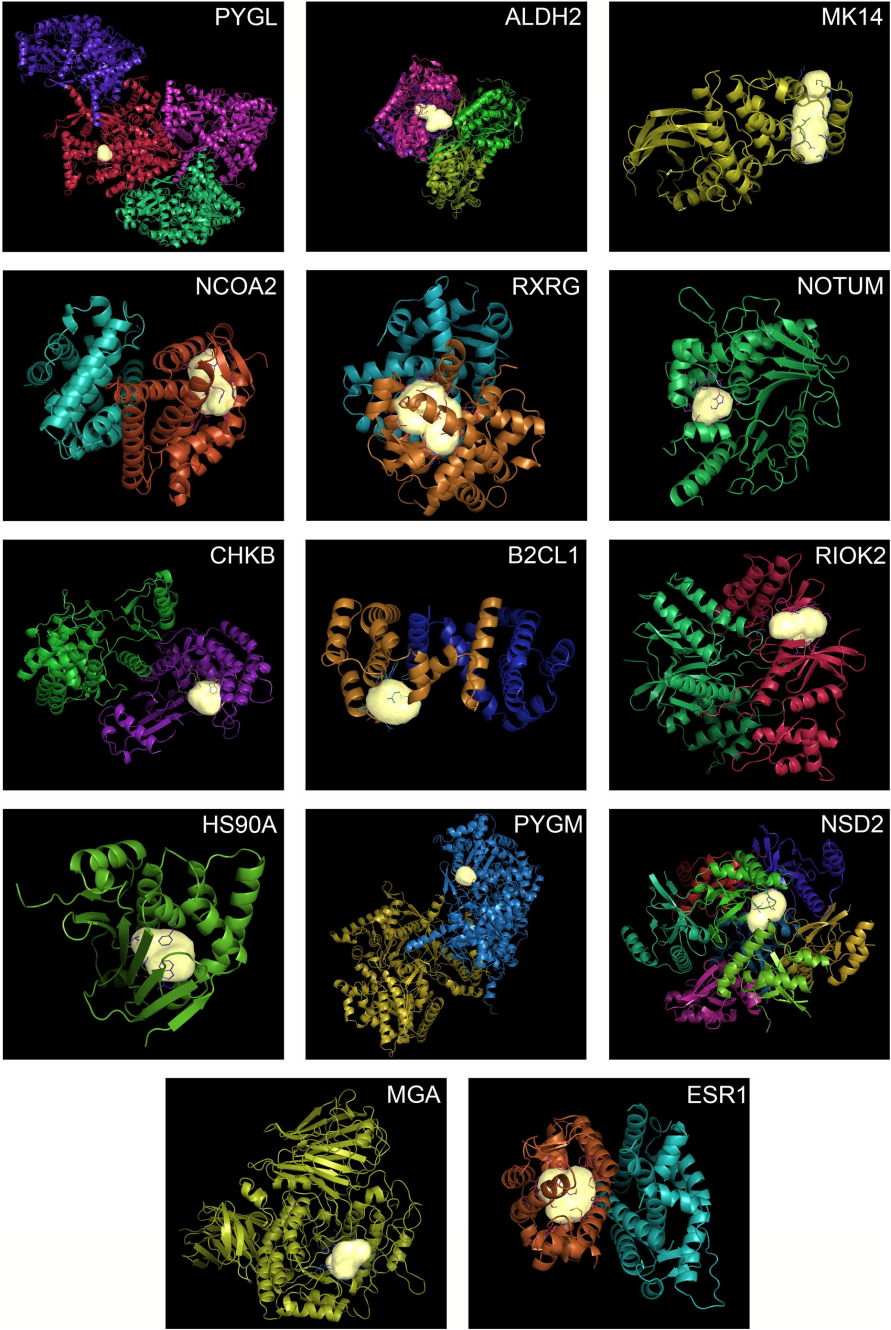
The 14 proteins with binding pockets with high affinity for *R*(−)MTZ. 2D images of 3D reconstructed protein complexes resulted in having high binding affinity with *R*(−)MTZ. 3D reconstructions were performed on BioGPS software.

Following the results of the previous section, we focused our attention on the restricted list of targets obtained for each MTZ enantiomer using the most stringent cut‐off parameters. Accordingly, we proceeded to the visual inspection of the pocket shape considering as the definitive list of bona fide target proteins the 16 and 14 proteins shown in Figure 3D,E. Using the same procedure as for the binding of *S*(+)MTZ and *R*(−)MTZ to SOS1 and RASH, we visualized the binding pockets on the target proteins that were occupied by each of the two MTZ enantiomers. Compared to the binding pocket of the SOS1: RASH complex, all the binding pockets retrieved appeared to have a compact shape, as shown in Figure 5 (16 protein targets) and 6 (14 protein targets) for *S*(+)MTZ and *R*(−)MTZ, respectively (Figure 6).

### 3.4 Pathway Enrichment Analysis

**FIGURE 7:**
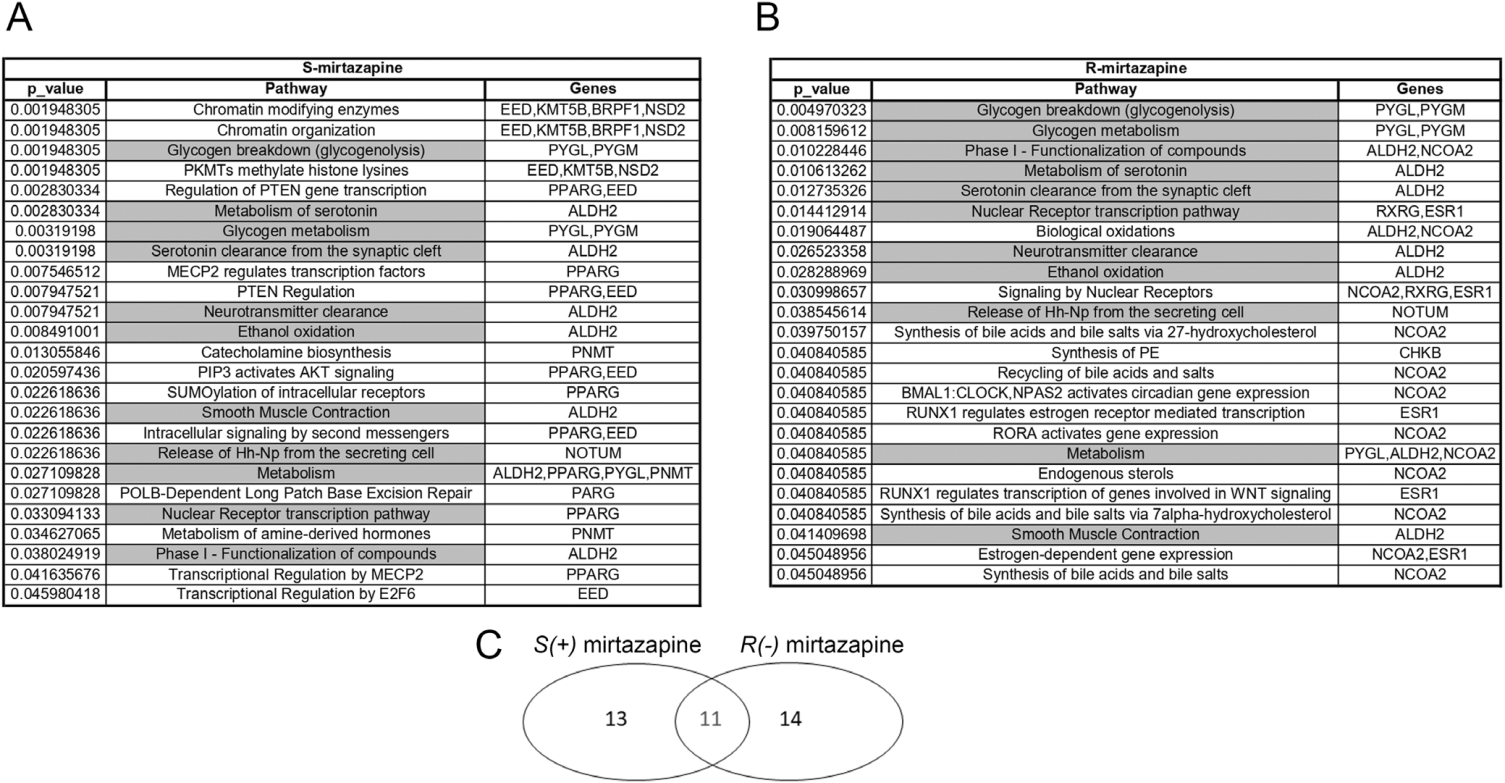
Enrichment analysis. (A) List of 25 enriched pathways for *S*(+)MTZ. In gray: 11 common pathways with *R*(−)MTZ. *p*‐value ≤ 0.05. (ZZscore ≥ 2.0; Zscore_poc ≥ 1.0; Zscore_mol ≥ 1.0; database: Reactome). (B) List of 24 enriched pathways for *R*(−)MTZ. In gray: 11 common pathways with *S*(+)MTZ. *p*‐value ≤ 0.05. (ZZscore ≥ 2.0; Zscore_poc ≥ 1.0; Zscore_mol ≥ 1.0; database: Reactome). (C) Venn diagram showing pathways enriched for only *S*(+)MTZ (14 pathways), only *R*(−)MTZ (13pathways) and both *S*(+)MTZ an *R*(−)MTZ (11 pathways).

To further investigate the mechanisms of action of mirtazapine, we performed a pathway enrichment analysis by using as input the 16 gene targets for *S*(+)MTZ and 14 gene targets for *R*(−)MTZ predicted with the more stringent parameters (ZZscore ≥ 2.0; Zscore_poc ≥ 1.0; Zscore_mol ≥ 1.0). Enrichment analysis was carried out using the *gost* function of the R package gprofiler2 (Easton et al. 2006) with the Reactome database as the source (see methods). As shown in Figure 7A,B, we obtained two independent lists of 25 and 24 enriched pathways for *S*(+)MTZ and *R*(−)MTZ, respectively. Of these, 11 pathways (in gray) were in common to both enantiomers, with a final list of 14 enriched pathways for *S*(+)MTZ only and 13 pathways for *R*(−)MTZ only (Figure 7A–C).

Remarkably, most of the pathways enriched for *S*(+)MTZ are involved in processes typically regulated by MECP2, the gene mutated in RTT, such as chromatin organization (2 pathways), epigenetic regulation (2 pathways), and transcription (6 pathways) including, most impressively, 2 pathways regulating either MECP2‐mediated translation or the translation of MeCP2 itself (Kolberg et al. 2020; Chen et al. 2015; Della Ragione et al. 2016). In addition, other notable findings relate to the pathways for neurotransmitter regulation, in particular of serotonin and catecholamine biosynthesis (4 pathways), which are known to be downregulated in RTT (Connolly and Zhou 2019). Finally, other groups of significant pathways include those related to metabolism and energy production through glycogen degradation (7 pathways) (Roux and Villard 2010), and intracellular signaling, including cascades downstream of neurotrophic receptors and sonic hedgehog (4 pathways) (Kyle et al. 2018). Finally, a pathway for smooth muscle contraction was alsofound.

Among the enriched pathways that are common between *R*(−)MTZ and *S*(+)MTZ, those related to glycogen degradation and metabolism, intracellular signaling, transcription, smooth muscle contraction, and neurotransmitter and serotonin regulation stand out. Instead, among the enriched pathways specific to *R*(−)MTZ (Figure 7B), the most prominent with respect to MeCP2 functions and RTT are those involved in the control of cholesterol and steroid hormone synthesis (7 pathways), lipid synthesis (1 pathway), circadian clock regulators (1 pathway), nuclear receptors activation (1 pathway) and Wnt signaling (1 pathway). These results are consistent with previous studies, which have recently reported that altered lipid and cholesterol biosynthesis is a hallmark of RTT (Pejhan and Rastegar 2021), that MeCP2 is involved in circadian clock regulation (Kyle et al. 2018) and that restoration of WNT signaling was beneficial in a RTT mouse model (Martínez de Paz et al. 2015). Importantly, the Ras/ERK pathway was not found among the enriched pathways, confirming that the stricter cut‐off parameters were more appropriate to avoid the collection of false‐positive targets.

### 3.5 OpenTargets Enrichment Analysis

**FIGURE 8:**
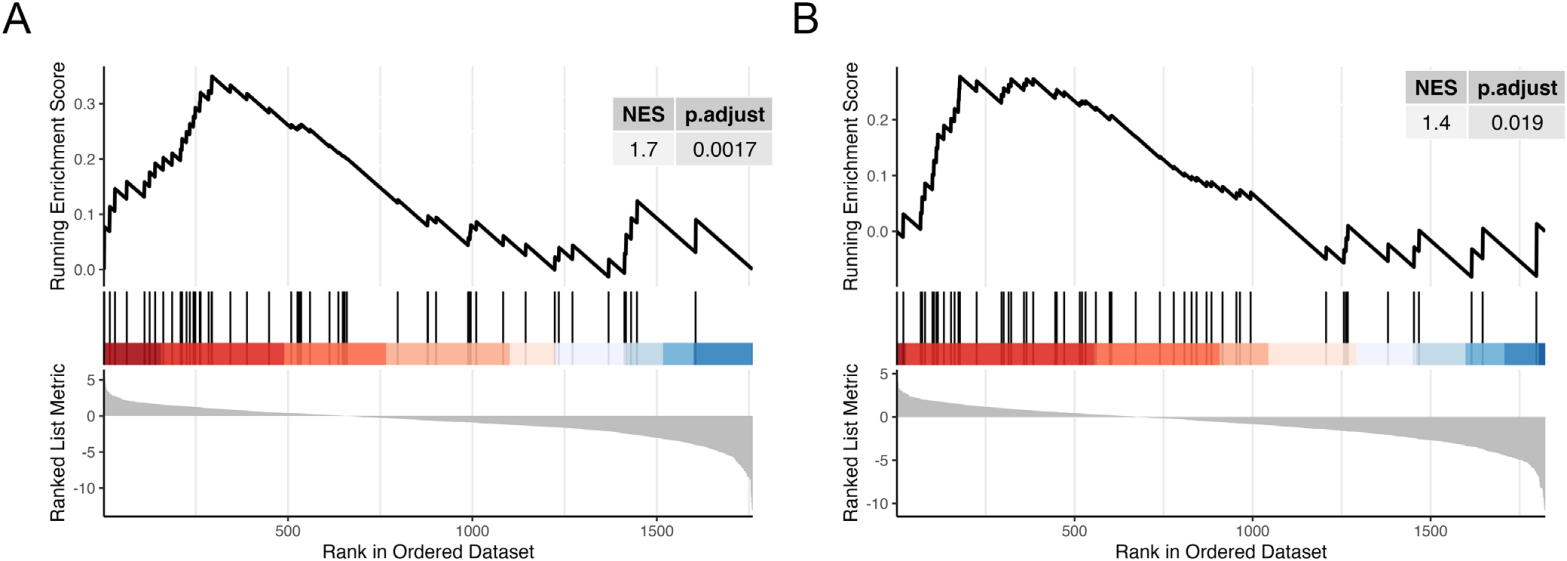
Gene set enrichment analysis on OpenTarget. GSEA plot reporting the targets enrichment obtained on the predicted lists for *S*(+)MTZ (A) and *R*(−)MTZ (B); targets are ranked by decreasing ZZscore values (bottom parts of the plots), and compared against the artificial pathway composed by Rett associated proteins retrieved from the OpenTargets platform (vertical black lines). In both cases, the table reports the obtained statistics: NES, that is, the normalized enrichment score, that reflects the degree to which a protein set is overrepresented at the top or bottom of a ranked list, and the adjusted *p*‐valued, obtained from 10 000 permutations tests.

To assess whether our analysis could effectively identify proteins associated with RTT, we used the OpenTargets database (Ghoussaini et al. 2021). OpenTargets is a platform that integrates human genetics and genomics data in order to systematically identify and prioritize potential drug targets selected from proteins linked to human diseases, including RTT. To evaluate the performance of our methods in prioritizing biologically relevant proteins associated with RTT, we sorted the input protein list based on the decreasing ZZscore values and performed GSEA on an artificial pathway containing proteins associated with RTT in the OpenTargets database. Notably, we observed significant enrichment for both mirtazapine enantiomers; Figure 8 shows the results for *R*(−)MTZ, with adjusted *p*‐value = 0.019 and NES = 1.4, and for *S*(+)MTZ, with adjusted *p*‐value = 0.0017 and NES = 1.7.

The “core enrichment,” which represents the proteins that are the main drivers of enrichment, contains for *S*(+)MTZ, the following proteins: **PPARG**, **HDAC6**, **SOS1**, KDM5A, **EZH2**, HMOX1, EHMT2, **MAP2**, EHMT1, **NQO1**, **BRD4**, NTRK1, **NCOR2**, **PDE4D**, NPC1, **FKBP5**, **BCL2**, NCOR1, AKT1, and GSK3B. For *R*(−)MTZ, the core enrichment includes the following proteins: **PPARG**, **BRD4**, **SOS1**, **HDAC6**, **PDE4D**, **FKBP5**, **EZH2**, **MAP2**, **BCL2**, KAT2B, **NCOR2**, HIF1A, BRD2, and **NQO1** (see list of abbreviations for full gene names). Interestingly, while 11 targets (marked in bold) appear in both lists, 8 targets are selective for *S*(+)MTZ (KDM5A, HMOX1, EHMT2, EHMT1, NTRK1, NPC1, NCOR1, AKT1 and GSK3B) and 3 targets are selective for *R*(−)MTZ (KAT2B, HIF1A and BRD2). This finding suggests that proteins associated with RTT are significantly enriched among the prioritized proteins, further confirming the biological relevance of the identified targets in the context under investigation and the value of the computational target ranking method developed in this study.

## 4 Discussion

In this study, we developed a novel in silico strategy combining double normalization (against protein pockets and inactive molecules) with stringent empirical thresholds to predict drug‐target interactions for mirtazapine S(+) and R(−) enantiomers. Our approach diverges from conventional ligand‐pocket affinity methods by introducing a comparative framework that prioritizes robustness over inclusivity. The selection of thresholds (ZZscore ≥ 2.0; Zscore_mol and Zscore_poc ≥ 1.0) was validated by the exclusion of false positives like SOS1/RASH in subsequent in vitro assays. Under these parameters, we identified 16 and 14 novel protein targets for *S*(+)MTZ and *R*(−)MTZ, respectively, with five shared targets. Pathway enrichment analysis further linked these targets to processes dysregulated in RTT, though notably not the Ras/Raf/MEK/ERK cascade—a result consistent with our experimental data and supporting the specificity of our thresholds.

### 4.1 Methodological Implications

This study represents the first application of BioGPS's ligand‐pocket predictions augmented by inactive‐drug normalization. While such computational pipelines are inherently sensitive to parameterization, our systematic comparison of relaxed versus stringent thresholds revealed two critical insights. First, a subset of high‐confidence targets (e.g., ALDH2, NOTUM) emerged irrespective of threshold stringency, suggesting that they warrant further experimental validation. Second, relaxed thresholds introduced biologically implausible candidates, underscoring the necessity of conservative parameters for hypothesis generation.

A second methodological innovation was our strict “liganded pocketome” construction. Despite the advent of AI‐predicted structures (e.g., AlphaFold), we restricted analyses to human crystallographic structures of human proteins that had been experimentally validated by high‐resolution X‐ray crystallography (less than 2.5 Å) with confirmed ligand binding. This conservative approach limited the overall target coverage but allowed for an increase in precision, resulting in a high confidence but nonexhaustive target space. Indeed, the combination of conservative criteria—namely, the utilization of protein crystal structures and the restriction of the analysis to the liganded pocketome—enabled the isolation of a small set of highly plausible target proteins. Future iterations could integrate predicted structures to mitigate this limitation.

### 4.2 Targets and Mechanistic Insights

A notable finding of this study is the identification of several novel putative targets for MTZ, which exhibit only partial overlap between its two enantiomers. The divergent target profiles of *S*(+)MTZ and *R*(−)MTZ align with their established pharmacological differences. Indeed, it has long been established that *S*(+)MTZ and *R*(−)MTZ possess different pharmacological properties, with the *S*(+) enantiomer being responsible for 5‐HT2 and α2‐receptor antagonism, and the *R*(−) enantiomer for 5‐HT3 receptors blockade (Hsu et al. 2020). However, given the recent challenges to the notion of its effective ability to bind to serotonergic receptors (Gillman 2006), there is a growing imperative to explore alternative targets. The results of this study indicate that *S*(+)MTZ and *R*(−)MTZ may interact with genes underlying six core processes known to be altered in RTT patients and animal models, of which two are shared between the two enantiomers.

The first of the six core processes identified for *S*(+)MTZ involves the target proteins EED, KMT5B, BRPF1, and NSD2, which are involved in chromatin organization and epigenetic histone modifications. Both chromatin and epigenetic functions are affected in RTT and other neurodevelopmental rare diseases and require MeCP2 as a key regulatory molecule (Kolberg et al. 2020; Chen et al. 2015; Della Ragione et al. 2016; Jefferson 1998). Among these target proteins, Embryonic Ectoderm Development (EED) serves as the structural backbone of the histone‐modifying Polycomb repressive complex 2 (PRC2), which maintains epigenetic transcriptional silencing through histone H3K27 methylation (Martinez‐Delgado and Barrero 2022). Clinically, EED mutations are associated with cancer and Cohen–Gibson and Weaver syndromes, both characterized by skeletal abnormalities and intellectual disability (Huang et al. 2023). Lysine Methyltransferase 5B (KMT5B), a methyltransferase targeting histone H3K4, promotes transcriptional activation and has been implicated as a risk gene in autism spectrum disorders (Bao et al. 2024). Intriguingly, female mice with KMT5B haploinsufficiency exhibit symptoms resembling RTT, though the protein's broader substrate specificity complicates mechanistic interpretations (Wang et al. 2001; Wickramasekara et al. 2021). Bromodomain and PHD Finger containing factor 1 (BRPF1), another epigenetic regulator, functions within the MOZ/MORF histone acetyltransferase complex to modulate chromatin structure, with mutations linked to Opitz‐Caveggia syndrome (Daks et al. 2022) and the Intellectual Development Disorder (Carlson and Glass 2014). Finally, the Nuclear Receptor Binding SET Domain Protein 2 (NSD2) is an H3K36 methyltransferase involved in epigenetic control of chromatin (Yang 2015; Kinoshita et al. 2024). Collectively, these data underscore the centrality of epigenetic dysregulation in RTT pathogenesis and the potential for MTZ to interact with these pathological mechanisms (He et al. 2024).

A second key process specific to *S*(+)MTZ centers on peroxisome proliferator–activated receptor gamma (PPAR‐γ or PPARG), a nuclear receptor controlling transcription of genes involved in intracellular signaling. PPARG is expressed in multiple tissues, including the brain, where it plays a crucial role in regulating cellular differentiation during development as well as carbohydrate, lipid, and protein metabolism (Pohodich and Zoghbi 2015). PPARG operates as a heterodimer with the retinoid X receptor (RXR), binding to DNA response elements upon activation by endogenous ligands such as free fatty acids, prostaglandins, Vitamin B3, and members of the eicosanoids family of arachidonic acid metabolites (Kota et al. 2005). Notably, MeCP2 physically interacts with PPARG to inhibit its activity, an interaction stabilized by Wnt signaling—a pathway negatively regulated by NOTUM carboxylesterase, a shared target of both MTZ enantiomers (Grygiel‐Górniak 2014; Kweon et al. 2016). In addition, the polycomb protein EED, an aforementioned *S*(+)MTZ binding target, is involved in the epigenetic control of PPARG, participating in the signaling cascade that modulates its functions (Bayle et al. 2021). The convergence of these mechanisms is highlighted by findings that PPARG activity is diminished in RTT models and that its pharmacological activation (e.g., via leriglitazone) rescues mitochondrial dysfunction and dendritic atrophy, paralleling the positive effects observed with *S*(+)MTZ (Nerli et al. 2020; Li et al. 2024).

The third interesting core mechanism is specific for *R*(−)MTZ and involves nuclear receptor coactivator 2 (NCOA2), a transcriptional regulator with histone acetyltransferase activity. NCOA2, also known as glucocorticoid receptor‐interacting protein 1 (GRIP1), steroid receptor coactivator‐2 (SRC‐2), or transcriptional mediators/intermediary factor 2 (TIF2), interacts with several nuclear receptors including the PPARG/RXR complex, and the estrogen receptor (ESR1)—both identified in this study as putative targets for *S*(+)MTZ. Notably, another novel target, specific for *R*(−)MTZ is the Retinoid X receptor gamma (RXR‐gamma, RXRG), also known as NR2B3 (nuclear receptor subfamily 2, group B, member 3), a nuclear receptor that responds to the ligands mentioned above and cooperates with NCOA2. This NCOA2's partnership with RXRG is of particular relevance for our study, as it drives oligodendrocyte maturation and myelination in the developing CNS (Musokhranova et al. 2023; Cullingford et al. 1998). Additionally, NCOA2 is known to interact with the ESR1, which also emerged as a putative target for *R*(−)MTZ in our in silico analysis. Furthermore, NCOA2 (with the alternative name SRC‐2) has been shown to coactivate the CLOCK‐BMAL1 heterodimer, a master regulator of circadian rhythms (Huang et al. 2011). This finding has suggested that NCOA2 is a positive regulator of the circadian clock, which is known to be disrupted in RTT (Stashi et al. 2014). Given the well‐documented sleep disturbances in RTT, the identification of circadian rhythm‐regulating proteins as novel MTZ targets is a key finding of this study that explains the improved sleep quality observed in patients treated with this drug (Flores Gutiérrez et al. 2020).

The fourth novel target progress is the phospholipid metabolism, which involves the enzyme choline kinase beta (CHKB; CKβ), a specific target for *R*(−)MTZ. CHKB catalyzes the synthesis of phosphatidylcholine (PC), a mitochondrial membrane phospholipid critical for neuronal plasticity (Li et al. 2015; Aoyama et al. 2004). CHKB deficiency disrupts mitochondrial function and is linked to muscular dystrophy, while PC supplementation restores membrane integrity in neurodegenerative contexts (Aoyama et al. 2004; van der Veen et al. 2017; Tavasoli et al. 2022; Mejia and Hatch 2016). In RTT, PC levels are reduced in cerebrospinal fluid, and *Mecp2*‐deficient neurons exhibit impaired PC turnover during growth phases—a defect that may contribute to stalled neurite extension (Magaquian et al. 2021; Zandl‐Lang et al. 2022). Although conflicting data exist regarding brain lipid profiles in RTT models (Viola et al. 2007), the observed therapeutic benefits of choline supplementation in mice, and their reversal by PC biosynthesis inhibitors (Seyfried et al. 2009), strongly implicate CHKB as a modulator of RTT pathophysiology and support the importance of our finding on the *R*(−)MTZ docking on CHKB.

As a fifth core process, we have identified as a new target for both MTZ enantiomers the production of energy through the mobilization of glucose‐1‐phosphate by the enzymes glycogen phosphorylases, specifically PYGM in muscle and PYGL in liver (Chin et al. 2019). The process mediated by PYGM is of particular importance during periods of elevated muscular activity, such as exercise. Additionally, PYGM is involved in insulin and glycogen signaling pathways, insulin resistance, and necroptosis and is not solely expressed in muscle tissue, but also in other tissues, including the brain, lymphoid tissue, blood, and ovaries (Chin et al. 2019). In the liver, glucose‐1‐phosphate is converted into glucose‐6‐phosphate and ultimately free glucose, which is released into the bloodstream to maintain blood sugar levels during fasting. Interestingly, both enzymes are expressed in glial cells (Migocka‐Patrzałek and Elias 2021). While the connection of PYGM/PYGL to RTT remains to be established, the beneficial effects of glycogen synthase kinase 3 (GSK3) inhibition in RTT models suggest that PYGM/PYGL modulation may offer therapeutic potential (Llavero and Zugaza 2024).

Lastly, the sixth potential target process, predicted for both *S*(+)MTZ and *R*(−)MTZ, concerns the oxidative pathway and lipid peroxidation, and involves the enzyme aldehyde dehydrogenase 2 (ALDH2). ALDH2 is a member of the aldehyde dehydrogenase enzyme family and is the second enzyme of the major oxidative pathway of detoxification of alcohol‐derived acetaldehyde in both the liver and the brain (Rizk et al. 2021). This enzyme has recently been implicated in the detoxification of aldehydes produced by lipid peroxidation and the degradation of neurotransmitters in the brain under increased oxidative stress, and has therefore been proposed as a novel neuroprotective therapeutic target in neurodegenerative diseases (Alnouti and Klaassen 2008). Remarkably, lipid peroxidation as a result of oxidative stress is a key pathological mechanism that has been described in RTT (Deza‐Ponzio et al. 2018).

The analysis of Rett‐associated targets with OpenTargets revealed very few targets (in total = 9) related to clinical data (ClinVar: MECP2, FOXG1, CDKL5, GABBR2, MIR718, IRAK1, DOK7, RHOBTB2, MAP2) and three connected to Reactome: MECP2, HDAC1, SIN3A. All the other reported targets have very low scores, and their review is beyond the scope of the present paper. BioGPS prioritized PPARG and SOS1 within the top RTT‐associated targets, but the latter was experimentally disproven. While OpenTargets provides a rapid, objective target ranking for understudied diseases like RTT, its sparse clinical data remain a limitation. Conversely, BioGPS is constrained by its reliance on experimentally determined protein‐ligand structures and excludes uncharacterized targets. Nonetheless, both platforms enriched for MTZ‐sensitive pathways. This suggests that their combined use—augmented by predicted protein structures—could refine future drug discovery pipelines. To our knowledge, this was the first investigation for a rare disease that compared an OpenTargets list with structure‐based predictions made by BioGPS (Valacchi et al. 2017).

### 4.3 Study Limitations and Future Directions

Four key limitations temper the interpretation of our findings. First, the reliance on crystallized, liganded pockets led to the exclusion of targets lacking structural data (e.g., GPCRs like 5‐HT2A). In fact, the in silico prediction results are biased by the availability of ligand‐bound 3D structures accessible in the PDB, which are determined via X‐ray crystallography at resolutions below 2.5 Å and deposited before 2022 (we used the version 22.02 of the BioGPS software from Molecular Discovery Ltd., whose pocket database contains protein structures up to 2021). Therefore, the background used in the calculations is a list of all the genes available when all pockets are considered. While this conservative filter enhanced prediction reliability, it omitted 17 of MTZ's 18 known targets, namely the 5HT1A, 5HT2A, 5HT2B, 5HT2C, and 5HT7 serotonergic receptors, the Alpha‐1A, Alpha‐2A, Alpha‐2C, Beta‐1, Beta‐2 adrenergic receptors, the D1, D2, and D3 dopaminergic receptors, the H1, H3 histaminergic receptors, and the Kappa Opioid and LPA1 receptor. Notably, among the 18 known targets of MTZ, only Beta‐2 adrenergic receptor (ADRB2) resulted in the list of 25 717 pockets. This pocket was lost with the further prioritization based on the ZZscore ≥ 2.0. Second, the inert drug set used for normalization, though chemically diverse, may incompletely represent the “null” pharmacological space. Third, an additional bias could arise from the different number of pockets that refer to different genes: this point was neither graphically nor mathematically addressed yet and will warrant further studies. Fourth, as with all in silico predictions, experimental validation remains essential—particularly for targets like NSD2 and CHKB, where mechanistic links to RTT are plausible but unproven.

Future studies could address these gaps by: (1) integrating AlphaFold‐predicted structures to expand the pocketome (https://alphafold.ebi.ac.uk/); (2) systematically optimizing inactive‐drug reference sets; and (3) employing orthogonal assays (e.g., thermal shift, in vivo binding) to validate top candidates.

## 5 Conclusions

In this study, we have proposed a novel strategy for the identification of novel targets and pathways following a drug repositioning experiment. Despite the inherent limitations, our dual‐normalization strategy advances in silico target discovery methods and could serve as a robust foundation for the identification of novel molecular targets for any drug of interest. The identification of noncanonical MTZ targets—particularly those tied to epigenetic regulation, lipid metabolism, and circadian modulation—provides a framework for understanding its clinical effects in RTT. More broadly, this approach is adaptable to drug‐repurposing efforts for other neurodevelopmental disorders with complex, multifactorial pathogenesis. Indeed, neurodevelopmental diseases are complex scenarios where a full understanding of the mechanism of action is very difficult to achieve compared to other cases (Valacchi et al. 2017). As structural biology and computational tools evolve, such pipelines will become increasingly important for translational hypothesis generation towards a deeper understanding of drug specificity (Santiago et al. 2017; Mulinari 2014). Furthermore, the method can be extended to other diseases for which phenotypic screening of a panel of drugs can be performed. This broadens the applicability of the approach beyond RTT, further assisting researchers in formulating hypotheses regarding the mechanisms of action of different drugs in different diseases.

## Author Contributions

**Ottavia Maria Roggero:** data curation, formal analysis, investigation, visualization, methodology, writing – original draft, writing – review and editing. **Nicolò Gualandi:** conceptualization, data curation, formal analysis, investigation, methodology, software, visualization, writing – review and editing. **Viviana Ciraci:** investigation, methodology, validation, writing – review and editing. **Vittoria Berutto:** investigation, validation, writing – review and editing. **Emanuele Carosati:** conceptualization, data curation, formal analysis, methodology, software, supervision, visualization, writing – review and editing, resources. **Enrico Tongiorgi:** conceptualization, funding acquisition, supervision, writing – review and editing, writing – original draft, resources, project administration.

## Conflicts of Interest

The authors declare no conflicts of interest.

### Peer Review

The peer review history for this article is available at https://www.webofscience.com/api/gateway/wos/peer‐review/10.1111/jnc.70093.

## Data Availability

The data that support the findings of the study are available from the corresponding authors upon request.
